# NEK Family Kinases: Structure, Function, and Role in Disease

**DOI:** 10.3390/biom15101406

**Published:** 2025-10-02

**Authors:** Brandon M. Baker, Julia R. Boehling, Sarah Knopf, Stephanie Held, Margarite Matossian, Jorge A. Belgodere, Van T. Hoang, Bridgette M. Collins-Burow, Elizabeth C. Martin, Sean B. Lee, Matthew E. Burow, David H. Drewry, Robert H. Newman

**Affiliations:** 1 Department of Biology, North Carolina A&T State University, Greensboro, NC 27411, USA; 2 Tulane Department of Medicine, Section of Hematology & Medical Oncology, Health Science Center, Tulane University, New Orleans, LA 70112, USA; jboehling@tulane.edu (J.R.B.); sknopf@tulane.edu (S.K.);; 3 Department of Microbiology, Immunology and Genetics, University of Chicago, Chicago, IL 60637, USA; 4 Tulane Cancer Center, Tulane University, New Orleans, LA 70112, USA; 5 Department of Biological and Agricultural Engineering, Agricultural Center, Louisiana State University, Baton Rouge, LA 70803, USA; 6 Department of Pathology & Laboratory Medicine, School of Medicine, Tulane University, New Orleans, LA 70112, USA; 7 Structural Genomics Consortium, UNC Eshelman School of Pharmacy, University of North Carolina at Chapel Hill, Chapel Hill, NC 27599, USA; 8 UNC Lineberger Comprehensive Cancer Center, School of Medicine, University of North Carolina at Chapel Hill, Chapel Hill, NC 27599, USA

**Keywords:** Never-in-Mitosis A-related kinase (NEK), understudied protein kinase, phosphorylation-dependent signaling, DNA damage response, inflammation, centrosome dynamics, cancer, neurodegenerative disorders, ciliopathies, drug discovery

## Abstract

The Never-in-Mitosis A-Related Kinase (NEK) family is an important, yet largely understudied, family of protein kinases involved in the regulation of a variety of critical cellular processes. Consequently, dysregulation of NEK function has been linked to the etiology and progression of several disorders, including cancer, ciliopathies, neurodegenerative disorders, inflammatory disorders, and other pervasive diseases. In this review, we have summarized recent findings to provide an overview of the NEK family and their diverse functions within various cellular contexts. In parallel, we have highlighted the emerging roles of NEK family members in human health, identifying potential therapeutic targets within the NEK family and exploring their potential for future clinical applications. Finally, we have addressed ongoing challenges and emerging research directions in this rapidly evolving field, aiming to pave the way for future discoveries and innovations.

## 1. Introduction

**Figure 1:**
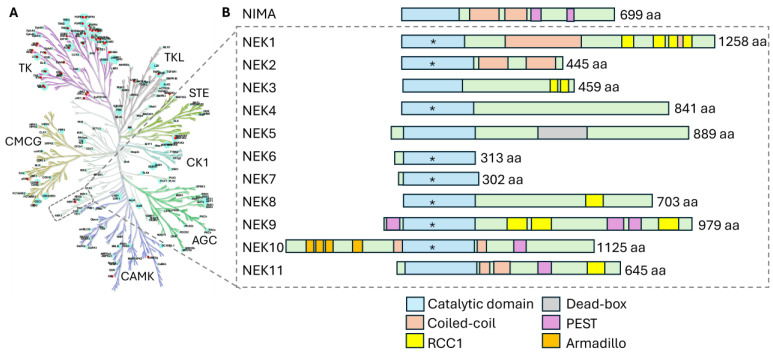
The NEK family. (**A**) The NEK family occupies a distinct branch on the kinome tree (dashed box). (**B**) Domain structure of NEK family members. A schematic representation highlighting the domain structure of NEK family members, including the catalytic domain (cyan), coiled-coil motif (salmon), DEAD-box domain (gray), PEST sequence (pink), regulator of chromosome condensation 1 (RCC1) repeats (yellow), and Armadillo repeats (orange). See text for details. Protein lengths are indicated in amino acids (adapted from [1]). Asterisks (*) represent NEK family members with a conserved regulatory tyrosine residue in the catalytic domain.

The Never-in-Mitosis A-related kinase (NEK) family contains eleven serine (Ser; S)/threonine (Thr; T) protein kinases that occupy a distinct branch on the human kinome phylogenetic tree (Figure 1A). NEK family members play important roles in diverse cellular processes, including cell cycle progression, primary cilia formation, centrosome dynamics, and the DNA damage response (DDR) [1]. As such, the functions of NEK family members are critical in maintaining cellular homeostasis across eukaryotic organisms.

The foundation for studying NEKs was established with the discovery of Never-in-Mitosis A (NIMA) in *Aspergillus nidulans*, a key mitotic kinase that regulates the G2/M transition [2]. This discovery led to the identification of the NEK family as a distinct group of kinases conserved from fungi to humans [3]. NIMA was shown to be essential for chromosome condensation and spindle organization, establishing the prototype for a new family of S/T kinases. Using the NIMA catalytic domain as a reference, the first mammalian NEKs—NEK1, NEK2, and NEK3—were identified in the early 1990s by homology-based cloning. These studies confirmed their conservation beyond fungi and revealed their localization to centrosomes and the cytoskeleton [4]. The expansion of the family accelerated in the late 1990s as expressed sequence tag (EST) projects uncovered NEK4 through NEK9 and the completion of the Human Genome Project enabled annotation of the final members, NEK10 and NEK11 [5,6]. Since then, functional genomics, proteomics, and CRISPR/Cas9 studies have revealed the roles of NEKs in diverse cellular processes—from cilia maintenance (NEK1, NEK8, NEK10) to tumor progression (NEK2, NEK6, NEK11)—cementing the NEKs as essential regulators of genomic stability and cellular architecture [6,7].

Although first discovered as NIMA in *Aspergillus*, phylogenetic evidence shows that the NEK family arose much earlier. A comprehensive analysis revealed that an ancestral NIMA-like kinase gene had already undergone several duplications in the last common ancestor of all eukaryotes—a ciliated cell that carried at least five *NEK* genes [8]. As eukaryotic lineages diverged and evolved increasingly complex ciliary structures, these ancestral NEKs diversified into distinct subfamilies containing homologous genes from both unikont lineages (e.g., mammals) and bikont lineages (e.g., green algae, ciliates). The ancestral NEKs likely coordinated cell cycle progression with cilia and centrosome function—a link that still defines much of their biology today.

Lineage-specific gene expansions further shaped this diversity. For example, while non-ciliated fungi like *Aspergillus* and yeast retain only a single *NEK* gene, multiciliated ciliates like *Tetrahymena* carry nearly 40 *NEK* genes [1]. Mammals evolved 11 distinct NEKs (NEK1–NEK11), with some retaining ancestral roles in the cell cycle and cilia, while others acquired novel functions. Evidence from phylogenetic clustering shows that modern NEKs group into several well-supported clades containing both unikont and bikont representatives, indicating that these subfamilies arose before the unikont–bikont split [8]. This ancient diversification laid the foundation for the functional breadth of the modern NEK family, spanning mitosis, centrosome regulation, cilia dynamics, and DNA repair.

Following this ancestral diversification, the 11 human NEKs further specialized through gene duplication events. One gene copy preserved the original role while the other diverged, enabling functional innovation without loss of essential activity [9]. While the conserved catalytic core remained stable, non-catalytic regions accumulated new interaction motifs and phosphorylation sites, expanding their regulatory capacity. As organisms became more complex, the NEKs adopted a “division of labor,” decreasing redundancy and sharpening control over cell cycle and structural processes. NEK1 and NEK3, for example, regulate cilia and centrosome integrity, while NEK6, NEK7, and NEK9 coordinate mitotic spindle dynamics [1]. By balancing conserved phosphorylation sites with lineage-specific regulatory motifs, NEKs adapted to the distinct signaling demands of different tissues and species. Given their central roles in maintaining genomic stability and cell cycle control, several NEKs are implicated in cancer and neurodegenerative disorders, highlighting their potential as therapeutic targets [10,11].

In humans, NEK family members (NEK1-NEK11) exhibit unique expression profiles and nonredundant roles in cellular regulation, including meiosis, ciliogenesis, and genome integrity. This functional diversity is closely tied to their structural complexity, as described in more detail below.

## 2. Structure-Function Relationships Among NEKs

### 2.1. The NEK Catalytic Domains

All NEKs share a conserved kinase domain that serves as the core around which other functional domains are organized (Figure 1B). The catalytic domain of NEK family members is an ~250–300 amino acid module responsible for ATP binding and phosphotransfer to protein substrates. In all NEKs, the kinase domain catalyzes phosphotransfer of the gamma (γ)-phosphate of ATP to either Ser or Thr phosphoacceptor sites on their target proteins. Interestingly, the only dual specificity kinase in the NEK family is NEK10, which can phosphorylate tyrosine (Tyr; Y) residues in addition to S/T sites. Across the NEKs, the core catalytic domain is approximately 40% identical to the founding *A. nidulans* NIMA kinase, underscoring their conserved enzymatic mechanism.

**Figure 2:**
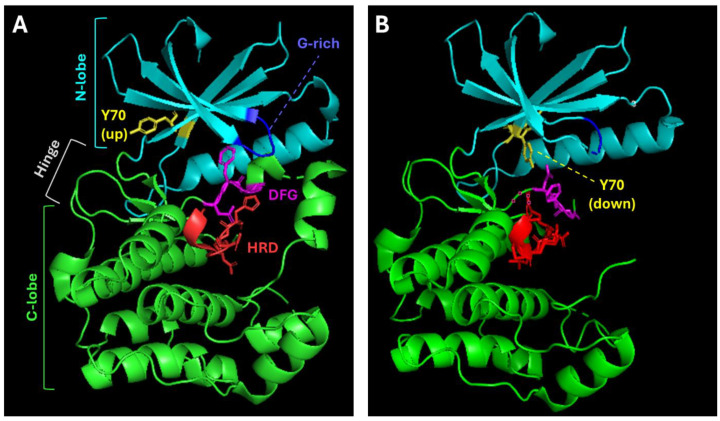
Conformational states of the NEK2 kinase domain. Ribbon diagrams of NEK2 in the (**A**) active, “Tyr-Up” conformation (PDB: 2W5H) and (**B**) the inactive, autoinhibited, “Tyr-Down” conformation (PDB: 2XK4). The hinge region located between the N-lobe (residues 1–89; cyan) and the C-lobe (residues 90–273; green) forms the ATP-binding cleft. Meanwhile, the G-loop (residues 17–20), positioned between the β1 and β2 strands in the N-lobe (blue), helps to stabilize ATP’s γ-phosphosphate. The catalytic loop (residues 135–145) contains the HRD motif (red sticks), while the activation loop extends beyond the DFG motif (magenta sticks). In the active state, the regulatory Tyr (Y70) (yellow stick) located near the αC/β4 loop adopts an “up” conformation, pointing outward and supporting proper alignment of the αC-helix. This facilitates the formation of a Lys-Glu salt bridge that is required for catalysis. In the inactive state (**B**), the catalytic loop and activation segment remain visible, but the activation loop is shifted inward compared to A. Notably, the regulatory Y70 is rotated into the active site, assuming a “down” conformation that disrupts the αC-helix alignment and prevents the Lys-Glu interaction. All structures were constructed in PyMol version 3.1.3.1 (Schrödinger, Inc., New York, NY, USA).

Similar to all eukaryotic protein kinases, the kinase domain in NEK family members adopts a bilobed fold composed of an N-terminal lobe (N-lobe) and a C-terminal lobe (C-lobe) (Figure 2A) [12]. The N-lobe, which is ~100 amino acids for most NEK family members, consists primarily of β-strands and the critical αC-helix. The αC-helix contains both the glycine-rich loop (corresponding to Gly-x-Gly-x-x-Gly in subdomain I) involved in γ-phosphate binding and the VAIK motif on the β3 strand that anchors α- and β-phosphates of ATP (Figure 2A). In contrast, the C-lobe, which is ~150 amino acids in most NEK family members, is composed almost exclusively of α-helices. Residing in this lobe are the HRDLKPEN catalytic loop (subdomain VI), which positions the substrate hydroxyl for phosphotransfer, and the aspartate-phenylalanine-glycine (DFG) motif, which maintains the proper alignment of the catalytic machinery during phosphorylation events (Figure 2A; subdomain VII). The activation segment (T-loop), whose conformation governs active versus inactive states, lies between the DFG and the alanine-proline-glutamate (APE) motifs [13]. Finally, the histidine-arginine-aspartate (HRD) motif plays an important role in coordinating phosphate groups during catalysis [12].

While the NEKs share many features in their catalytic domain with other eukaryotic kinases, they also possess distinctive features that are unique to the family. For instance, a key lysine residue (corresponding to K33 in NEK1) located in the ATP-binding pocket helps to anchor ATP in the active site through electrostatic interactions [14]. Similarly, specific residues within the activation loop can influence the catalytic efficiency of NEK family members. For instance, activation of NEK6 and NEK7 requires phosphorylation of S206 and S195 in their T-loops, respectively [5]. Meanwhile, NEK2 contains a cluster of three phosphorylatable residues (T170, S171, T175) in its activation segment [15]. Though phosphorylation of T175 appears to be most crucial, even a single Glu to Asp phosphomimetic substitution at either T170, S171, or T175 enhances NEK2 activity. Though it is not currently clear whether all three residues must be phosphorylated to achieve full activity, this may suggest a multi-site phosphorylation mechanism in NEK2, where multiple negative charges in the loop may cooperatively stabilize the active conformation.

Interestingly, except for NEK3, NEK5, and NEK11, all NEK family members also contain a conserved Tyr residue within the active site. A corresponding Tyr residue is found in only ~10% of the rest of the human kinome [16]. This residue acts as an inhibitory switch that adopts different conformations to regulate NEK enzymatic activity [1]. Crystal structures of NEK2 and NEK7 in the active and inactive states have enhanced our understanding of the structural basis for this regulatory mechanism. For instance, the crystal structure of NEK7 reveals a ‘Tyr-down’ conformation, where Tyr97 blocks the alignment of catalytic residues needed for ATP binding and subsequent phosphorylation of substrates (Figure 2B) [17]. This Tyr occupies a position at the top of the hydrophobic regulatory spine (R-spine) in the kinase N-lobe (near the αC-β4 loop), blocking the proper alignment of catalytic residues and preventing substrate access [12,17]. This arrangement keeps NEK7 in an inactive state, effectively acting as a molecular switch. NEK2 contains Tyr70 in an equivalent position while NEK6 contains Tyr108, both of which are predicted to play a similar regulatory role [1]. In the case of NEK9, displacement of this Tyr residue through dimerization or interaction with its noncatalytic tail causes a conformational change that relieves autoinhibition and permits activation-loop autophosphorylation [17,18]. Interestingly, other NEK family members also display dimerization-coupled activation mechanisms. For instance, NEK2 homodimerizes via its coiled-coil domain (see Section 2.2.1 below; Figure 1B) to enable trans-autophosphorylation, whereas NEK6/7 rely on NEK9 to promote a “back-to-back” kinase-domain association that dislodges the autoinhibitory Tyr [17]. These structural details have been crucial in designing NEK selective inhibitors, which leverage these inactive conformations to achieve both specificity and efficacy.

### 2.2. Accessory Domains in NEK Family Members

Though the catalytic domain is a unifying feature within the NEK family, each member exhibits unique substrate preferences and regulatory mechanisms that lead to their specialized roles in various cellular contexts. Figure 1B highlights the domain structures of NEK family members, including the catalytic domain, coiled-coil motif, DEAD-box domain, PEST sequence, regulator of chromosome condensation 1 (RCC1) repeats, and Armadillo (ARM) repeats. These modular domains confer specialized regulation–from controlling protein stability to directing subcellular localization–and thereby tune NEK activities in processes such as cell cycle progression, centrosome/cilia function, and microtubule organization (Table 1). Below, we examine each of these domains, detailing their molecular function, examples in specific NEKs, and how they modulate kinase activity or cellular roles.

#### 2.2.1. Coiled-Coil Domain

Coiled-coil motifs augment NEK signaling by driving kinase assembly, enabling auto-transactivation (e.g., for NEK2), stabilizing NEK associations with centrosomal or cytoskeletal structures, and potentially organizing higher-order complexes necessary for processes like spindle formation and ciliogenesis. Coiled-coil motifs are structural domains formed by intertwined α-helices, often featuring heptad repeats (e.g., (abcdefg)_n_) with hydrophobic residues at positions a and d. They serve as dimerization or oligomerization interfaces, mediating a wide range of protein–protein interactions, assembly of multiprotein complexes, and higher-order structures [19]. In many proteins, the presence of a coiled-coil domain promotes homodimer formation or heteromeric binding to other coiled-coil proteins, which can be critical for their function. Importantly, NEK oligomerization mediated by coiled-coil domains can create a surface to recruit substrates or regulators.

Most NEK family members have coiled-coil regions, which are typically located in the C-terminal tail outside the kinase domain [3]. For instance, the *A. nidulans* NIMA kinase contains a C-terminal coiled-coil motif that mediates oligomerization, and this feature is broadly conserved among its mammalian counterparts. In fact, of all human NEKs, only NEK4, NEK6, and NEK7 lack predicted coiled-coil motifs. For instance, NEK2 has a well-characterized leucine zipper, which is a specific type of coiled-coil motif, in its C-terminus. Likewise, NEK1, NEK3, NEK5, NEK8, NEK9, NEK10, and NEK11 all have coiled-coil regions of varying lengths, often overlapping with other regulatory elements [3]. Interestingly, NEK10 has two putative coiled-coil motifs flanking its kinase core. Together with its other protein binding motifs (see below), NEK10’s coiled-coil motifs allow it to act as a scaffold inside the cell. Similarly, the coiled-coil domain of NIMA kinase is believed to facilitate cooperativity in its mitotic functions.

The coiled-coil domains also critically influence NEK activity and localization by promoting assembly of kinase complexes. For example, the leucine zipper in NEK2 is required for its homodimerization, which in turn is necessary for its autophosphorylation and full activation [20]. Disruption of this dimer interface (e.g., by deletion of the coiled-coil domain) prevents NEK2 activation.

Besides activation, coiled-coil domains also help target NEKs to specific organelles by increasing the avidity of dimers for specific docking sites. For instance, the coiled-coil domain of NEK2 contributes to its localization at centrosomes, where it phosphorylates centrosomal proteins to trigger centrosome disjunction [21,22]. Interestingly, the coiled-coil domain found in NEK2A (but not the other NEK2 isoforms) also has a nucleolar retention role.

#### 2.2.2. PEST Sequences

PEST sequences are specific amino acid motifs prominently enriched in proline (P), glutamic acid (Glu; E), S, and T residues. These unique amino acid compositions confer structural characteristics that signal proteins for rapid degradation by the ubiquitin-proteosome pathway [23]. Therefore, proteins containing PEST domains typically exhibit short half-lives due to their susceptibility to ubiquitin-dependent proteolysis, a key mechanism maintaining cellular protein homeostasis and regulating diverse biological processes. For instance, proteins involved in cell cycle transitions often have PEST sequences, ensuring precise degradation timing that is necessary for proper cell division [24,25]. Similarly, many PEST-containing proteins are involved in signaling processes that require rapid turnover to swiftly adapt cellular responses to external cues [26,27].

Functionally, PEST domains play critical roles in modulating NEK activity by tightly regulating protein abundance and conferring precise temporal control throughout the cell cycle. For instance, the NIMA kinase from *A. nidulans* has two C-terminal PEST domains that are essential for its destruction immediately following mitosis [3]. Timely degradation of NIMA via PEST-dependent ubiquitination is vital for mitotic exit and cell cycle progression.

Five of the eleven mammalian NEK family kinases—NEK1, NEK3, NEK9, NEK10, and NEK11—possess putative PEST sequences in their non-catalytic regions (Figure 1B) [28,29]. These PEST sequences impact NEK function in various ways. For instance, deletion of NEK3’s PEST sequences in neurons leads to distorted neuronal morphology with disrupted polarity and deacetylation of microtubules via HDAC6 [30]. Similarly, Fin1, a NIMA homolog in fission yeast, exhibits analogous PEST motifs at its C-terminus. Deletion of the PEST domains leads to excessive Fin1 accumulation, lethal chromatin condensation, and disrupted mitotic timing in yeast [31].

Interestingly, while human NEK2 lacks a PEST domain, its expression levels throughout the cell cycle appear to be regulated through ubiquitin-dependent degradation in a manner similar to its PEST-containing counterparts. For instance, the predominant NEK2 splice variant, NEK2A, contains a KEN box and a D-box, which are recognized by the multicomponent E3 ubiquitin ligase, anaphase promoting complex/cyclosome (APC/C) [22]. Poly-ubiquitylation of NEK2A directs its proteasomal degradation upon mitotic completion. Conversely, the shorter NEK2B isoform lacks these domains, allowing it to remain stable following mitosis by evading poly-ubiquitination [29].

#### 2.2.3. DEAD-Box Domains

DEAD-box domains, which are named after the highly conserved D-E-A-D amino acid motif, represent a distinctive structural feature characteristic of a family of RNA helicases [32]. These domains possess the ability to bind RNA and hydrolyze ATP, which allows them to unwind RNA duplex structures or facilitate remodeling of RNA-protein complexes. Consequently, DEAD-box helicases play important roles in regulating gene expression, including governing mRNA processing, ribosome assembly, RNA nuclear export, and translational regulation. Their activity promotes dynamic conformational changes in RNA molecules, thereby functioning as key regulators across diverse RNA metabolic pathways [33].

Within the human NEK family, NEK5 uniquely harbors a putative DEAD-box helicase-like domain, represented as an ~200 amino acid segment within its primary structure [16]. Structurally, the NEK5 kinase domain resides at the N-terminal region, whereas the predicted DEAD-box domain is located at its C-terminus (Figure 1B). This structural arrangement suggests that the DEAD-box helicase-like domain might function as a regulatory tail that affects the kinase activity and/or subcellular targeting of NEK5 [34,35].

The presence of a DEAD-box domain in NEK5 may also indicate roles in RNA or ribonucleoprotein (RNP) biology. Although definitive substrates and specific cellular roles for NEK5 are not fully elucidated, the DEAD-box helicase motif potentially enables NEK5 to engage directly with RNA molecules or associated RNP complexes. This domain may position NEK5 strategically at sites involved in RNA processing or stress response assemblies such as stress granules, thereby influencing RNA metabolism during critical cell-cycle stages or cellular stress conditions [35].

Furthermore, recent studies have uncovered mitochondrial localization of NEK5, suggesting its role in the maintenance of mitochondrial homeostasis [35]. However, whether the DEAD-box domain contributes directly to these mitochondrial functions has not been established. Given known roles of DEAD-box helicases in RNA-mediated regulatory networks, the NEK5 DEAD-box domain might also serve as an adapter or scaffold, connecting kinase signaling pathways to RNA processing events or responding to RNA-derived cellular signals. The mechanistic link integrating kinase signaling cascades with RNA metabolism could facilitate coordinated regulation of cellular responses to genomic stress or metabolic perturbations [36].

Collectively, the presence of a DEAD-box helicase domain suggests broad functionality of NEK5, from mediating cell-cycle control and stress response signaling to RNA metabolism. This hypothesis is supported by reports of functional interplay between DEAD-box RNA helicases and diverse kinase pathways, as well as the recognized involvement of NEK5 in DDR mechanisms [34,36].

#### 2.2.4. Regulator of Chromosome Condensation 1 (RCC1)-like Domain

RCC1-like domains consist of tandem RCC1 repeats ~50 amino acids long that fold together, typically as a β-propeller, to mediate protein–protein interactions [37]. The archetypal RCC1 protein functions as a guanine nucleotide exchange factor (GEF) for the Ran GTPase, exchanging Ran-bound GDP for GTP [38]. RCC1-like domains are known to bind chromatin and establish a Ran-GTP gradient in the nucleus, playing crucial roles in chromosome condensation, spindle assembly, and nucleocytoplasmic transport [37,39]. These domains can serve as regulatory modules that target proteins to chromosomes or facilitate interactions with Ran and other factors.

In humans, both NEK8 and NEK9 contain stretches of tandem RCC1 repeats in their C-terminal regions [40,41]. In fact, this characteristic earned NEK9 its initial name, Nek-RCC1 (Nercc1), reflecting its hybrid kinase-RCC1 architecture [41]. The NEK9 N-terminal kinase domain (which is ~300 amino acids in length) is followed by an ~600 amino acid C-terminal region composed of RCC1 repeats [41]. Similarly, NEK8, a kinase implicated in ciliopathy, harbors an RCC1-repeat region [40]. The RCC1 domains in the NEKs are highly conserved with respect to the canonical RCC1, thus they are presumed to fold in a manner similar to Ran-GEF [42]. Since other NEKs lack this domain, the ability to interact with Ran-GEF or chromatin-associated machinery appears to be unique to NEK8 and NEK9.

The RCC1-repeat domain serves regulatory and localization functions for NEKs. For instance, in NEK9, the RCC1-like tail acts as both an intramolecular inhibitor and a scaffold by binding the kinase domain to maintain autoinhibition while also binding the Ran GTPase [43]. This Ran binding activity suggests NEK9 might localize to chromatin or respond to Ran’s nucleotide state during mitosis, although NEK9 itself does not function as a Ran-GEF [44]. Additionally, the NEK9 RCC1-like domain is crucial for downstream signaling processes, providing a docking interface for the mitotic NEK6/NEK7 kinases. The long C-terminal RCC1-repeat region of NEK9 directly interacts with NEK6 and NEK7, forming a kinase cascade complex [45], relieving autoinhibition, and facilitating full activation of NEK6/NEK7 [3]. This process is central to mitotic spindle assembly, as active NEK6/NEK7 downstream of NEK9 ensure proper spindle formation and cytokinesis in dividing cells [44].

Though the function of the RCC1-like domain in NEK8 is less well characterized, it may similarly regulate NEK8 kinase activity and/or its subcellular targeting [40]. Alternatively, given the role of NEK8 in cilia and centrosome stability, its RCC1-like repeats could tether NEK8 to nuclear or centrosomal structures or mediate interactions with Ran to regulate ciliogenesis. Together, the RCC1-like domains in NEK8 and NEK9 provide a Ran-linked regulatory module. In the case of NEK9, it enforces autoinhibition and serves as a scaffold for a mitotic kinase complex, illustrating how this domain connects NEK signaling to chromosome- and microtubule-associated events such as spindle assembly via Ran and downstream NEK6/7 [43].

#### 2.2.5. Armadillo Repeat Domains

Armadillo (ARM) repeats are tandem 42-amino-acid motifs that fold together into a superhelix, creating an elongated surface for protein binding [46]. ARM repeat domains (first characterized in the Armadillo protein of Drosophila melanogaster, which is homologous to mammalian β-catenin) serve as versatile protein–protein interaction platforms [47]. They often facilitate the assembly of multiprotein complexes by binding specific peptide sequences on target proteins. ARM-repeat proteins play a role in diverse cellular processes, ranging from transcriptional regulation (e.g., β-catenin’s ARM domain binds transcription factors) to intracellular transport (e.g., importins with ARM repeats bind cargo proteins). Thus, ARM domains are often associated with proteins that can act as a scaffold or an adapter, anchoring other proteins via its repeat surface.

Within the NEK family, NEK10 is notable for containing a cluster of four ARM repeats in its N-terminal region [48]. These repeats, together with coiled-coil segments, flank NEK10’s centrally positioned kinase domain [49]. The presence of ARM repeats makes NEK10 the most structurally divergent NEK family member, as it has the capacity for extensive protein–protein interactions via the ARM domain. Orthologs of NEK10 in other organisms, such as the *C. elegans* NEK-like 4 (NEKL-4), also carry ARM repeats and have been linked to ciliary structures, suggesting a conserved function for this domain [48].

Its four ARM repeats allow NEK10 to function as a scaffold, likely targeting it to specific complexes or subcellular regions. Similar to known ARM-repeat proteins, NEK10 ARM repeats can bind partner proteins to regulate its kinase activity or to bring them into close proximity [50]. Computational analysis predicts that NEK10 ARM repeats mediate binding to specific proteins. For example, an in silico docking analysis found that NEK10’s ARM domain likely interfaces with the small heat-shock protein, HSPB1, with specific residues in the ARM region contacting HSPB1 [51]. Given HSPB1’s involvement in stress and cytoskeletal stability, such interactions suggest NEK10 may be a part of a stress-responsive complex [51]. Additionally, the ARM-containing N-terminus of NEK10 may target it to cilia or centrosomal structures [52]. For instance, human NEK10 has been implicated in motile cilia function with mutations causing bronchiectasis due to defective airway cilia [48]. Therefore, the ARM repeat module could recruit NEK10 to the base of cilia or to axonemal protein complexes critical for ciliogenesis. More generally, NEK10 is known to mediate a UV-induced G2/M checkpoint and activate mitogen-activated protein kinase (MAPK)-dependent signaling upon stress [3,48]. In this context, the ARM domain may help assemble the necessary signaling proteins.

## 3. Regulation of NEKs

The structural diversity among NEK family members has important implications for their regulation and cellular function. The regulation of NEK family members is achieved through a variety of mechanisms, with phosphorylation playing a central role. For instance, the phosphorylation status of NEKs plays a key role during their activation and helps to dictate many of their cellular functions. Additionally, subcellular localization and interactions with binding partners are key factors that influence the activity and specificity of NEKs. For example, at the end of G2 phase, NEK2 localizes to the centrosome, where high local concentrations of the kinase drive its dimerization and subsequent autophosphorylation [15,53]. These processes are closely regulated during the cell cycle to synchronize its activity with mitotic events, thereby ensuring accurate centrosome separation and spindle assembly during mitosis. Meanwhile, NEK1 is heavily involved in DNA repair processes, interacting with proteins such as ataxia telangiectasia and Rad3-related kinase (ATR) to maintain genomic stability [35].

The RCC1-like domain in NEK8 and NEK9 and the ARM motifs in NEK10 also contribute to the functional specialization of these kinases [40,41,54,55]. Specifically, the tandem RCC1-like repeats in NEK8 and NEK9 act as both localization and activation modules [40,41,55]. In the case of NEK9, Ran-GTP-mediated recruitment to spindle poles in late G2 phase drives kinase-domain dimerization and exposes the activation loop for PLK1- and CDK1-dependent phosphorylation [41]. Meanwhile, docking interactions at the ciliary base via its RCC1-like domain positions NEK8 for Aurora kinase A (AURKA)-mediated phosphorylation [40]. Phosphorylation relieves an autoinhibitory interaction between the NEK8 C-terminal RCC1-like cluster and the kinase domain, thereby licensing NEK8 to regulate polycystin-2 trafficking and ciliary length.

The NEK10 ARM domain serves a dual autoinhibitory/scaffolding role. In unstressed cells, the N-terminal ARM repeats pack against the kinase domain, masking the substrate-docking groove and preventing unwarranted activity [1,35]. Upon genotoxic stress, the master regulator of the DDR pathway, ataxia telangiectasia mutated (ATM), phosphorylates a short linker just N-terminal to the ARM array, triggering a conformational “release” that both exposes the active site and creates a new docking surface for the mitogen-activated protein kinase kinases (MAP2Ks), MEK1/2 [35]. This assembly enables rapid phosphorylation of extracellular signal regulated kinase 1 and 2 (ERK1/2), directly coupling the DNA-damage pathway to a cytoplasmic checkpoint effector cascade [54].

In addition to their activation profiles and subcellular localization, the cellular functions of NEK family members are also regulated at the level of substrate selection. For instance, van de Kooij et al. recently used oriented peptide library screening (OPLS) to identify the preferred phosphorylation motif for every human NEK family member except NEK11 [16]. These studies revealed several interesting trends. For example, NEK family kinases exhibited a general preference for either leucine (Leu; L), methionine (Met; M), phenylalanine (Phe; F), or tryptophan (Trp; W) at the −3-position relative to the phosphosite (i.e., three residues N-terminal to the phosphosite). Each of the NEKs also preferred Trp at the −4-position, which is unique among the human kinases, and selected against a proline (Pro;P) residue at the +1-position, which distinguishes the NEKs from other kinases involved in cell cycle regulation such as cyclin-dependent kinases (CDKs) and MAPKs [16].

In addition to these general preferences, each NEK displayed unique substrate preferences [16]. For example, NEK1, 3, 4, 5, and 8, collectively termed Group 1/3/4/5/8, preferentially phosphorylated Thr over Ser, with strict selection for hydrophobic residues (L/M/F/W) at the –3 position and basic residues such as arginine (Arg; R) or lysine (Lys; K) at the –2 position. In contrast, Group 2/6/7/9 family members preferred to phosphorylate Ser residues, with acidic residues such as aspartate (Asp; D) or glutamate (Glu; E) at the –2 position. The selection of a Ser or Thr phosphoacceptor site by Group 2/6/7/9 and Group 1/3/4/5/8, respectively, appears to be related to the presence of either a Leu (NEK2, 6, 7, 9) or an isoleucine (Ile; I) (NEK1, 3, 4, 5, 8) residue in the DFG + 1 position [16]. Interestingly, Group 2/6/7/9 family members also appeared to prefer a phosphotyrosine residue one residue C-terminal to the phosphosite, which may suggest some degree of mitotic crosstalk. Finally, NEK10, a dual-specificity kinase, exhibited a consensus motif similar to the canonical NEK motif when phosphorylating Ser sites. However, when targeting Tyr, an additional requirement for an aromatic or hydrophobic residue at the +1 position immediately C-terminal to the phosphosite was observed.

Group 1/3/4/5/8 members are further distinguished by their shared preference for a positively charged Arg residue at the −1 position. NEK1 and NEK4 also prefer a basic residue (R/K) in the +2 position but deviate from the general NEK consensus motif in that they show little to no selectivity for Trp at the −3 position [16]. Similarly, NEK3 exhibits a preference for hydrophobic residues at both the –3 and +1 positions.

The two primary NEK groups (i.e., Group 1/3/4/5/8 and Group 2/6/8/9) can be further sub-divided into four specificity groups based on their phosphoacceptor site and other molecular determinants surrounding the phosphosite [16]. For instance, members of specificity group 1, which consists of NEK1, NEK3, and NEK4, preferentially phosphorylate Thr residues flanked by an Arg residue at the −1 position, Likewise, NEK1 and NEK4 also exhibit a clear preference for a basic residue (R/K) at the +2 position and do not show a strong preference for Trp at the −3 position. The preferred motif of selectivity group 2 members, which comprises NEK5 and NEK8, is similar to that of selectivity group 1 members, expect they show little preference for Arg at the −1 position. Meanwhile, selectivity group 3 kinases, which consist of NEK2 and NEK10, are distinguished from selectivity group 1 and 2 family members by their preference for Ser as the phosphoacceptor site. They generally exhibit a preference for F/L/M in the −3 position as well as a hydrophobic residue in the +1 position and an Arg residue in the +2 position [16]. Finally, like selectivity group 3 kinases, the selectivity group 4 members, NEK6, NEK7, and NEK9, exhibit a strong preference for Ser as the phosphoacceptor site. However, OPLS experiments also suggest that the substrate preferences of selectivity group 4 kinases diverge substantially from those of other NEK family members. For instance, unlike the other human NEKs, NEK6, NEK7 and NEK9 display a preference for negatively charged residues at the −5, −4, and −2 positions, with a particularly pronounced selectivity for Asp at the −2 position relative to the phosphosite. NEK6 and NEK7 share 85% identity within their catalytic domains, and this similarity is reflected in further overlap in their respective consensus phosphorylation motifs [16]. Indeed, both NEK6 and NEK7 prefer hydrophobic residues at the −3 and +1 positions, particularly Leu at –3. Interestingly, the selectivity group 4 NEKs are known to form a functional circuit during mitotic progression and spindle formation, where NEK9 serves as an upstream activator of NEK6/7 activity [41]. Together, their unique substrate preferences, coupled with distinct regulatory mechanisms and activation profiles, allow NEK family members to regulate distinct and diverse biological functions, as discussed in more detail below.

## 4. Functions of Specific NEK Family Members

**Figure 3:**
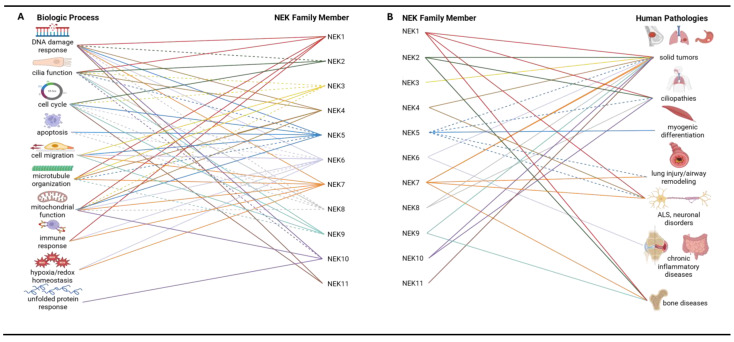
NEK family members play diverse roles in health and disease. The eleven NEK family members have been linked to the regulation of key biological process (**A**) and the etiology of various human pathologies (**B**). Solid lines represent a direct role in the given biological process or disease while dashed lines represent a potential role in the corresponding biological process/disease.

NEK family members play important roles in the regulation of diverse cellular processes, including mitosis, DDR, ciliary function, and the immune response (Figure 3A). Consequently, dysregulation of NEKs has been implicated in pathologies ranging from cancer to neurological disorders to chronic inflammation (Figure 3B). Below, we discuss the known cellular functions of each NEK family member and their role in disease.

### 4.1. NEK1

NEK1 plays a pivotal role in ciliary maintenance and repairing DNA double-strand breaks, a process crucial for preserving genomic stability and preventing oncogenic mutations (Figure 3; Table 2) [56]. For instance, among the NEKs, NEK1 is one of the earliest responders to DNA double-strand breaks (DSBs). Following exposure to ionizing radiation or oxidative stress, Tousled-like kinase 1 (TLK1) phosphorylates NEK1 on T141 within its kinase domain, causing a marked increase in NEK1 activity and promoting its rapid redistribution to γH2AX-positive nuclear foci [57]. This TLK1→NEK1 step occurs independently of the activation of the canonical phosphoinositide-3-kinase (PI3K)-like kinases, ATM/ATR, and is essential for proper checkpoint activation and damage sensing [58].

Once activated, NEK1 phosphorylates key mediators of the ATR-CHK1 axis. NEK1 targets ATR interacting protein (ATRIP), stabilizing the ATR–ATRIP complex and priming ATR for autophosphorylation (Table 3) [59]. This, in turn, allows efficient CHK1 activation and enforcement of the intra-S and G2/M checkpoints. In parallel, NEK1’s C-terminal coiled-coil domain binds homologous recombination factors such as radiation-sensitive 54 protein (RAD54) and the meiotic recombination 11 protein (MRE11)-radiation-sensitive 50 (RAD50)-Nijmegen breakage syndrome protein 1 (NBS1) complex, likely leading to their phosphorylation and coordinating DNA end resection and repair pathway choice [60].

In support of this role, genetic knockout or siRNA-mediated knockdown of NEK1 leads to profound DDR defects. For instance, NEK1-deficient HeLa cells exhibit persistent γH2AX foci long after DNA damage [93]. Likewise, they fail to arrest the cell cycle properly and accumulate chromosomal aberrations and aneuploidy. Consistently, expression of a non-phosphorylatable NEK1(T141A) mutant recapitulates these phenotypes and impairs ATR/CHK1 signaling following oxidative insult, underscoring the necessity of NEK1’s kinase activity and its TLK1-mediated activation for genomic stability [57].

Interestingly, structural analysis suggests that NEK1 undergoes substantial conformational changes in response to DNA-damaging agents like cisplatin [93]. To probe these dynamics in a near-native setting, researchers have combined in-cell labeling with gentle, post-lysis purification and cell-conditioned crystallography. In the hydrogen-deuterium exchange mass spectrometry (HDX-MS) workflow, living cells are pulse-labeled with D_2_O following cisplatin treatment, after which NEK1 and its bound partners are rapidly immunopurified under quenched, low-pH conditions before being proteolyzed and analyzed by LC-MS to map changes in solvent accessibility that occurred in situ [93]. Complementing these studies, high-resolution “snapshots” have been obtained by isolating endogenously modified NEK1 from drug-treated mammalian cells, preserving physiological phosphorylation and cofactors, and crystallizing the kinase domain alone or co-crystallized with short peptides from ATR, BRCA1, or MDC1 to lock in DNA-damage-activated conformations [13,62].

These combined approaches have revealed that the NEK1 kinase domain can adopt distinct conformations depending on the cellular context, particularly after DNA damage [62]. Interestingly, these shifts appear to actively modulate NEK1 interactions with key DDR effectors such as ATR, BRCA1, and MDC1. Disruption of these interactions, whether through mutations in NEK1 or its partners, leads to genomic instability and cancer progression [94]. This suggests that NEK1 functions as a central hub that, through dynamic conformational rearrangements, either promotes DNA repair or triggers cell death in accordance with the severity of the DNA damage. To capture these conformational and interaction dynamics in their native context where HDX-MS and crystallography fall short, Gregorcyzk and colleagues combined highly specific immunoprecipitation of endogenous NEK1–C21orf2 complexes with size-exclusion chromatography on native lysates, preserv- ing physiologically relevant states [62]. They then applied AlphaFold modeling to predict how disease-linked mutations perturb the binding interface and validated those predictions in gene-edited knockout (KO) cell lines. These studies suggested that reintroducing wild-type NEK1 restored both ciliogenesis and homologous recombination. This integrative workflow dissected NEK1 conformational regulation and tied structural remodeling directly to cellular phenotypes. This dynamic behavior may allow NEK1 to act as a central hub in coordinating the cellular response to DNA damage, interacting with a variety of proteins to either promote DNA repair or trigger cell death, depending on the severity of the damage.Recent studies have also uncovered the involvement of NEK1 in neuroinflammation and neuronal survival, particularly in the context of neurodegenerative diseases (Figure 3; Table 4). For instance, mutations in NEK1 have now been implicated in amyotrophic lateral sclerosis (ALS), Parkinson’s disease (PD), hereditary spastic paraplegia (HSP), and cerebellar ataxia, underscoring its multifaceted role in neuronal homeostasis [9,62,95,96,97,98]. In ALS, heterozygous loss-of-function (LoF) variants in NEK1 account for ~2–3% of all cases [96]. For example, in a Chinese ALS cohort, 2.6% of patients were found to carry NEK1 variants, with many of these mutations being novel LoF variants [95]. These mutations contribute to the disease by exacerbating DNA damage, impairing mitochondrial function, and accelerating motor neuron death. For instance, patient-derived motor neurons harboring NEK1 LoF mutations accumulated γH2AX foci and chromosomal breaks due to failure to initiate the early stages of DDR [9]. Proteomic and functional analyses suggest that NEK1 interacts with both microtubule and nucleocytoplasmic transport machinery such that its haploinsufficiency destabilizes microtubules and impairs nuclear import, thereby compromising axonal transport and protein homeostasis [95].

Concurrently, NEK1 forms a complex with C21orf2 to regulate primary ciliogenesis. To this end, ALS-linked variants trigger a Ca^2+^–Aurora A–HDAC6 cascade that drives ciliary disassembly, α-tubulin hypoacetylation, and mitochondrial fragmentation defects that are fully rescued by HDAC6 inhibition [62,96]. Notably, NEK1 mutations have been shown to affect mitochondrial dynamics, leading to increased apoptosis, hypersensitivity to genotoxic agents, and altered mitochondrial processes such as reduced mitophagy and increased reactive oxygen species (ROS) production [99].

In PD, NEK1 deficiency disrupted retromer-mediated lysosomal degradation of the A20 ubiquitin regulator, sensitizing cerebrovascular endothelial cells to RIPK1-dependent apoptosis and necroptosis [97]. This compromised blood–brain barrier (BBB) integrity, amplified neuroinflammation, and accelerated α-synuclein aggregation in Nek1^−^/^−^ mice. The disruption led to increased neuroinflammation and facilitated the aggregation of α-synuclein in neurons and other neural cells, a hallmark of PD pathology. Moreover, genetic studies have identified specific NEK1 polymorphisms, such as rs66509122, that are associated with a reduced risk of PD, suggesting a protective role for this variant [100]. Together, these findings suggest that NEK1 supports neural survival through the modulation of DNA repair, mitochondrial function, and the neuroinflammatory response, linking it to conditions like ALS and PD [101].

However, it is important to note that while NEK1 has been implicated in these disorders, additional studies are required to better understand its role in the pathology of these disorders. For instance, though genetic studies have strongly linked LoF variants in NEK1 to ALS, the evidence remains largely correlational. A significant gap in the field is the lack of a clear mechanistic understanding of how NEK1 haploinsufficiency leads to motor neuron death. Future studies using genetically precise animal or organoid models are essential to determine whether the observed defects in DNA repair, mitochondrial function, or ciliogenesis are the primary drivers of pathology or secondary consequences of cellular stress. Furthermore, it is unclear why motor neurons are particularly vulnerable to the loss of NEK1 function when it is expressed ubiquitously.

Interestingly, in addition to ALS and PD, NEK1 has also been implicated in several ciliopathic neurological disorders. For instance, mutations in NEK1 have been linked to HSP, cerebellar ataxias, and the rare genetic disorders, Joubert syndrome and Meckel-Gruber syndrome [96]. In HSP, NEK1 haploinsufficiency led to defective primary cilia and impaired Sonic hedgehog (Shh) signaling in corticospinal neurons, perturbing neuronal polarity and contributing to the characteristic axonal “dying-back” degeneration. Disruption of ciliary signaling in adult Purkinje neurons precipitated dendritic atrophy and motor incoordination, phenocopying human spinocerebellar ataxia.

NEK1 involvement in ciliary biology first became apparent when LoF mutations were shown to disrupt centrosome integrity, a prerequisite for primary cilium assembly [96]. Researchers demonstrated that NEK1–deficient cells fail to form normal primary cilia, instead exhibiting centrosome disassembly and absent axonemes [102]. Building on these studies, researchers localized NEK1 to the basal-body region of the centrosome in serum-starved retinal pigment epithelial 1 (RPE1) cells [96]. They found that siRNA-mediated depletion of NEK1 resulted in the production of aberrant, branched cilia. These data strongly suggested that the NEK1 coiled-coil domain plays a crucial role in stabilizing axonemal microtubules during ciliogenesis [96,100,102].

Mechanistically, NEK1 is believed to be activated via autophosphorylation during primary cilia formation. This leads to cell cycle–regulated stabilization of NEK1 at the basal body and promotes axoneme extension and proper ciliary signaling [102,103]. Interestingly, NEK1 interaction with the centriolar protein, C21orf2, adds another layer of control. For instance, C21orf2 recruits NEK1 to distal appendages where NEK1-mediated phosphorylation of C21orf2 allows it to evade FBXO3-mediated ubiquitylation and subsequent degradation, thereby modulating centrosome stability, ciliogenesis, and cell-cycle progression (Table 3) [63]. Consistently, mouse models bearing kat and kat2J Nek1 alleles recapitulate human ciliopathic phenotypes of progressive polycystic kidney disease (PKD), dwarfism, male sterility and craniofacial anomalies with renal epithelial cells displaying unusually long or multiple primary cilia [104]. These in vivo observations suggest that NEK1 is essential not only for cilium formation but also for maintaining ciliary length and number in a tissue-specific context.

At the molecular level, NEK1 stability is coordinated with the cell cycle by the APC–Cdc20 ubiquitin ligase [105]. When NEK1 escapes degradation, it antagonizes the CP110–CEP97 complex at the centrosome to promote axoneme extension and preserve ciliary architecture. Clinically, recessive NEK1 variants, such as G145R and L253S, have been identified in oral–facial–digital syndrome type II and short-rib thoracic dysplasia, directly linking NEK1 dysfunction to human ciliopathies and highlighting its conserved role in developmental signaling and tissue homeostasis [13,94,100].

During primary cilium formation, NEK1 undergoes dynamic re-localization and activation to orchestrate the assembly of this specialized organelle. Upon serum starvation and entry into G0/G1 arrest, NEK1 translocates from the cytoplasm to the basal body region and along the nascent axoneme, positioning it to phosphorylate substrates that promote axoneme elongation and structural stability. Its catalytic “on” switch is triggered by autophosphorylation of a conserved Thr residue within the activation loop, inducing conformational changes that enable full kinase activity [102]. Mutations that block this modification phenocopy NEK1 loss and lead to defective ciliogenesis [62]. Concurrently, the C-terminal interaction domain of NEK1 binds C21orf2 at the basal body. Formation of this NEK1–C21orf2 complex is essential for both ciliary assembly and downstream Hedgehog signaling [102]. Interestingly, NEK1 levels are dosage-sensitive: both kinase overexpression and deficiency impair cilia formation, underscoring the necessity for tightly regulated NEK1 expression throughout the primary cilium cycle [62].

Beyond its roles in neurodegenerative diseases and ciliopathies, NEK1 has also been implicated in various cancers, where it appears to exert a dual function promoting tumorigenesis on one hand while offering a potential avenue for radiosensitization on the other [56,106,107]. For instance, NEK1 overexpression has been observed in several malignancies, including gliomas, where its expression level correlates with tumor grade and poor patient prognosis [106]. Yeast-two-hybrid screening further revealed a broad NEK1 interactome encompassing 14-3-3 isoforms, ATRX, MRE11, p53 binding protein 1 (53BP1) and the protein phosphatase 2A (PP2A) regulatory subunit B56, highlighting its role in coordinating checkpoint signaling and phosphatase activity [93].

In glioma cells, NEK1 drives proliferation and survival through a multifaceted DDR network in which TLK1 phosphorylates NEK1 on T141, fostering its interaction with the ATR–ATRIP complex and priming ATR autophosphorylation at T1989 for efficient CHK1 activation [107]. Upon genotoxic stress, NEK1 is redistributed to γ-H2AX- and MDC1-positive foci, coordinating early damage sensing and checkpoint initiation [108]. NEK1 also binds the non-homologous end-joining (NHEJ) factor, Ku80, to promote end-joining and safeguard S-phase progression [9]. Concurrently, NEK1 scaffolds FANCD2 at interstrand crosslinks, enhancing its monoubiquitinylation and chromatin recruitment while phosphorylating RAD54 at S572 to stabilize replication forks and orchestrate RAD51 filament turnover during homologous recombination (Table 3) [13,61]. Like the ciliopathies discussed previously, NEK1 association with C21orf2 links DNA repair proficiency to ciliary signaling axes, highlighting a complex signaling hub that underpins both tumor growth and potential radiosensitization strategies [13].

In bladder cancer, NEK1 is part of a twelve gene tumor score panel used to predict the progression from non-invasive to muscle-invasive disease [109]. NEK1 also displays potential as a prognostic marker, with higher NEK1 expression levels being associated with more aggressive tumor behavior. In pancreatic cancer, NEK1 is similarly associated with poor prognosis, suggesting that it may play a role in the aggressive nature of this cancer [13].

NEK1 has also been identified as a promising target for radiosensitization, particularly in the context of fractionated radiotherapy [108]. By knocking down NEK1 expression, researchers observed increased sensitivity of cervical cancer cells to radiation-induced DNA damage, effectively delaying tumor growth in vivo [110]. This radiosensitization effect is believed to be due to role of NEK1 in DDR, such that its inhibition disrupts the repair of radiation-induced DNA breaks, leading to increased cell death. The broader implications of these findings are significant, as they suggest that targeting NEK1 could improve the therapeutic index of radiotherapy by selectively enhancing the radiosensitivity of tumor cells while sparing normal tissues. This approach could be particularly valuable in treating cancers that are resistant to conventional radiotherapy, offering a new avenue for improving patient outcomes.

However, the dual role of NEK1 presents a therapeutic paradox. While inhibiting NEK1 can sensitize cancer cells to radiation by crippling their DNA repair capacity, this strategy could have unforeseen consequences given its protective role in the nervous system. A critical challenge will be to develop strategies that can selectively inhibit NEK1 in tumor tissues or to identify a therapeutic window where the benefits of radiosensitization outweigh the risks of neurotoxicity.

### 4.2. NEK2

NEK2 plays an important role in centrosome dynamics, spindle assembly, and chromosome segregation during mitosis (Figure 3; Table 2) [111]. For instance, NEK2 facilitates the disassembly of the inter-centriolar linker, enabling centrosomes to separate so that they can establish a functional mitotic spindle [68]. NEK2 activity is tightly regulated by Polo-like kinase 1 (PLK1), another key S/T kinase that controls various stages of the cell cycle, including centrosome maturation and spindle assembly [112]. Specifically, PLK1 phosphorylates mammalian sterile 20-like kinase 2 (MST2), a component of the Hippo pathway, during the early stages of mitosis. MST2 phosphorylation prevents protein phosphatase 1 (PP1) from binding to the MST2-NEK2 complex, which releases NEK2 from its inactive state by promoting its autophosphorylation and allowing it to transition to the active form.

Once activated, NEK2 facilitates centrosome separation by regulating the disassembly of the inter-centriolar linker, which includes proteins such as C-Nap1 and rootletin [113]. NEK2-mediated phosphorylation of these proteins alters their structural integrity, promoting their displacement from the centrosome (Table 3) [55,111]. This process is critical for centrosome separation, which ensures the formation of a bipolar spindle—a structure necessary for accurate chromosome segregation and genomic stability [65,111].

In parallel, NEK2 contributes to spindle assembly checkpoint (SAC) signaling by localizing to the kinetochore, where it interacts with proteins such as mitotic arrest-deficient protein 1 (MAD1) and highly expressed in cancer 1 (HEC1) [66]. Specifically, NEK2-mediated phosphorylation of HEC1 on S165 enhances its interaction with MAD1 at kinetochores (Table 3). This interaction promotes stable accumulation of the MAD1–MAD2 complex on unattached or improperly attached chromosomes [114]. This, in turn, catalyzes conversion of MAD2 into its active, “closed” conformation, driving assembly of the mitotic checkpoint complex (MCC) composed of MAD2–CDC20–BUBR1–BUB3 and potent inhibition of the APC/C [115]. The net effect is sustained spindle assembly checkpoint signaling and a delay in anaphase onset until all kinetochores achieve proper microtubule attachments [114,116]. Promotion of SAC signaling, particularly on misaligned chromosomes, ensures that cells do not prematurely progress through mitosis before proper chromosome attachment [114,116]. Thus, targeting the NEK2-HEC1 interaction has emerged as a promising therapeutic strategy, particularly in cancers where SAC signaling is compromised. Together, these interactions are critical for ensuring correct microtubule attachments to kinetochores and facilitating accurate chromosome segregation. Thus, disruption of the NEK2/HEC1/MAD1 axis can induce mitotic arrest, leading to chromosomal instability in cancer cells [117].

In addition, NEK2 influences microtubule organization by phosphorylating centrosomal proteins like Nienin-like protein (NLP) and centrobin and modulating their roles in microtubule anchoring and stabilization, respectively, during mitosis [67,68]. For instance, by phosphorylating NLP during mitotic entry, NEK2 triggers NLP removal from the centrosome, allowing for the reorganization of the microtubule network necessary for spindle formation (Table 3) [67]. Similarly, NEK2-mediated phosphorylation of centrobin alters its microtubule-stabilizing function, ensuring the proper dynamics of the mitotic spindle (Table 3) [68]. This regulation is crucial for mitotic progression.

Beyond its established role in cell cycle progression, recent studies have uncovered a role for NEK2 in ATR-mediated DDR pathways, suggesting that it functions as a dual regulator of genomic stability and cell division. Indeed, NEK2 overexpression is strongly linked to tumorigenesis [7,35,118]. For instance, the overexpression of NEK2 has been correlated with poor prognosis in malignancies such as breast, ovarian, and lung cancer [118]. Consequently, NEK2 represents a promising target for cancer therapy, with small molecule inhibitors being developed to diminish its activity in disease states. NEK2 inhibitors aim to impair centrosome separation and mitotic progression, leading to cell cycle arrest and apoptosis in cancer cells [119]. For example, small-molecule inhibitors targeting NEK2 have demonstrated efficacy in disrupting centrosome dynamics, leading to mitotic arrest and apoptosis in cancer cells [120,121]. Notably, these inhibitors have shown potential in overcoming chemoresistance in drug-resistant cancers.

Despite its promise as a cancer target, the fundamental role of NEK2 in mitosis raises significant concerns about toxicity in healthy proliferating tissues, such as bone marrow and the intestinal epithelium. The central challenge for NEK2 inhibitors is to achieve a therapeutic window that allows for the killing of cancer cells (which are often more dependent on a properly functioning mitotic apparatus) without causing unacceptable side effects. Furthermore, the mechanisms of resistance to NEK2 inhibition remain largely unexplored and will be critical to address for any clinical advancement.

### 4.3. NEK3

NEK3, which functions at the crossroads of estrogen receptor signaling, the oxidative stress response, and intracellular transport, phosphorylates substrates that impact cytoskeletal organization, cell migration, and differentiation [30]. Recent findings also connect NEK3 to broader cancer signaling pathways, cardiovascular function, and neuronal health, revealing its role in development and disease progression [70].

Like NEK1 and NEK2, NEK3 plays a key role in regulating microtubule dynamics during intracellular transport and mitosis. In this context, one of the primary functions of NEK3 is to modulate the activity of acetyltransferases and deacetylases that regulate the acetylation status of α-tubulin, which directly impacts microtubule stability [30,121,122]. For instance, Zhang et al. found that mutations in NEK3 in patients with abnormal cardiac left-right patterning led to increased expression of the deacetylase, Sirtuin (SIRT2), resulting in the deacetylation of α-tubulin [123]. Deacetylation of α-tubulin, in turn, compromises microtubule stability, leading to abnormal cardiac left-right patterning in laterality disorders such as situs inversus and heterotaxy [124]. This regulation contributes to cell shape and supports vital cellular functions like intracellular transport, where microtubules serve as tracks for the movement of organelles and other components within cells [123]. However, the precise molecular mechanisms by which NEK3 regulates microtubule acetylation remain unclear. While studies show that NEK3 mutations can alter the expression of deacetylases like SIRT2, it is not known if this is a direct regulatory event or an indirect downstream consequence. Identifying the direct substrates of NEK3 that mediate its effects on the cytoskeleton is a critical next step to validate it as a potential target for controlling cancer cell motility.

Interestingly, some of the observed effects may be related to the influence of NEK3 on nucleocytoplasmic transport through the nuclear pore complex (NPC). Although its role in NPC assembly remains incompletely understood, recent findings suggest that NEK3 indirectly impacts NPC stability by regulating nucleoporins [26]. Nucleoporins are critical components of the NPC, which governs the transport of molecules between the nucleus and cytoplasm [125]. Indeed, in addition to alterations in SIRT2 expression, Zhang et al. found that knockdown of NEK3 also led to the downregulation of inner ring nucleoporins, including NUP205, NUP188, and NUP155 [123]. The disruption in NEK3 activity, along with reduced NUP205 levels, may compromise NPC integrity and hinder nuclear-cytoplasmic transport, further implicating NEK3 as a candidate gene for ciliopathies.

In the context of mitosis, the regulatory role of NEK3 is very important. Microtubules form the mitotic spindle, which is necessary for the segregation of chromosomes during cell division. NEK3 influences the assembly and function of miotic spindles, ensuring proper cell cycle progression and minimizing the risk of chromosome missegregation [124]. This process is fundamental to maintaining genomic stability since disruptions in NEK3 activity can lead to errors in chromosome segregation, potentially contributing to the progression of cancer [123]. This also has important implications for processes like spindle assembly and DNA replication, further connecting NEK3 to key mitotic dysfunctions.

Beyond its roles in microtubule dynamics, NEK3 is also involved in various signaling pathways involved in breast cancer progression, including prolactin signaling, estrogen receptor signaling, Rho GTPase signaling, cell cycle regulation, and PI3K/AKT signaling [118]. For instance, NEK3 overexpression has been linked to increased cell motility and invasiveness in breast cancer and prostate cancer, traits that are essential for cancer metastasis [70]. This is especially evident in the interaction between NEK3 and the guanine nucleotide exchange factor, VAV2 (Table 3). This interaction modulates signaling through the human prolactin receptor and plays a key role in prolactin-mediated processes in breast cancer. NEK3-mediated phosphorylation of VAV2 enhances cytoskeletal reorganization and cell motility, which increases the invasive potential of cancer cells, further implicating NEK3 in the promotion of oncogenesis [118].

### 4.4. NEK4

One of the primary functions of NEK4 is to maintain genomic integrity following exposure to DNA damaging agents. For instance, by recruiting the DNA-dependent protein kinase (DNA-PK) to sites of DNA damage, NEK4 has emerged as an important regulator of NHEJ during DNA repair [9]. This function also links NEK4 to cell cycle arrest and checkpoint control, highlighting the role of NEK4 in preventing the propagation of genetic mutations [3,126].

Interestingly, recent studies also point toward NEK4 involvement in critical processes that drive cancer progression, such as the epithelial–mesenchymal transition (EMT), mitochondrial dynamics, and the sensitivity of cancer cells to chemotherapy [127]. For example, NEK4 plays an important role in regulating EMT, a process where epithelial cells lose their adhesion and polarity and take on mesenchymal characteristics that make them more mobile and invasive [128]. In lung adenocarcinoma cells, NEK4 acts as a promoter of EMT by regulating the activity of suppressor of mothers against decapentaplegic 3 (SMAD3) and zinc finger E-box binding homeobox 1 (ZEB1) during transcriptional growth factor beta (TGF-β)-induced EMT [127]. Moreover, when NEK4 is knocked down, SMAD3 activity drops, leading to reduced EMT and increased E-cadherin levels [129]. Additionally, Ding and colleagues reported that knockdown of NEK4 increased the expression of E-cadherin and inhibited the tumor formation in murine models of lung adenocarcinoma [127].

In addition to its roles in DDR, NEK4 has emerged as a key regulator of mitochondrial dynamics and bioenergetics. NEK4 localizes to the outer mitochondrial membrane, where it promotes ERK1/2-dependent phosphorylation of the cytosolic GTPase, dynamin-related protein 1 (DRP1), on S616, stabilizing DRP1 oligomerization and inducing membrane constriction to drive fission [126]. In parallel, overexpression of NEK4 led to increased expression of the adaptor, mitochondrial fission factor (MFF), which is believed to enhance DRP1 recruitment and complex stabilization. Once at the membrane, NEK4 helps trigger MEK1/2-mediated activation of ERK1/2 activation, as evidenced by increased phosphorylation at T202/Y204. In this way, it creates a positive feed-forward loop that amplifies DRP1 phosphorylation on S616 and sustains robust mitochondrial division.

This highly coordinated fission process not only increases the number of discrete mitochondrial units, which boosts respiratory capacity and enhances the efficiency of ATP production, but it also primes damaged fragments for selective autophagic clearance [126]. By fragmenting dysfunctional segments into smaller units, NEK4-driven fission facilitates PINK1- and Parkin-dependent mitophagy, preventing the accumulation of ROS and preserving organelle quality. Under stress conditions, excessive fragmentation lowers membrane potential and promotes cytochrome c release, sensitizing cells to apoptotic stimuli via caspase activation [130]. Finally, during mitosis, NEK4-dependent promotion of fission ensures equitable mitochondrial distribution to daughter cells, safeguarding energy homeostasis and cellular function across cell generations [126]. Thus, by regulating mitochondrial fission, NEK4 ensures the proper distribution and quality control of mitochondria, which is essential for maintaining cellular homeostasis and preventing the accumulation of damaged mitochondria that could trigger apoptosis.

Mitochondria also play a key role in controlling cell death through pathways like intrinsic apoptosis and necroptosis [131]. During apoptosis, cellular stress signals trigger the release of cytochrome c from the mitochondria into the cytosol [132]. This event initiates the formation of the apoptosome, which activates caspases and ultimately leads to cell death. The mitochondrial membrane’s integrity is vital to this process. Proteins like Bcl-2-associated X protein (Bax) and Bcl-2 homologue antagonist/killer (Bak) form pores that facilitate cytochrome c release [132]. NEK4 plays an important role in regulating these pathways by influencing mitochondrial dynamics and sensitizing cells to apoptosis [126]. Moreover, NEK4 has also been implicated in the extrinsic apoptosis pathway, where its inhibition results in a decrease in the expression of Survivin (also known as BIRC5), an anti-apoptotic protein [129]. Since Survivin is known to prevent apoptosis by inhibiting caspase activation, NEK4 inhibition may render cancer cells more susceptible to therapies like TRAIL that induce apoptosis [126,133].

Intriguingly, NEK4 has also been identified as a modulator of microtubule poison-induced cell death [134]. For instance, cells with deficiencies in NEK4 are characterized by impaired G2/M arrest and decreased formation of mitotic-like asters following Taxol treatment. Taxol, also known as paclitaxel, is a chemotherapeutic agent widely used in cancer treatment due to its ability to stabilize microtubules [135]. Stabilization prevents the normal breakdown of microtubules during cell division, effectively halting the cell cycle and leading to cell death, particularly in rapidly dividing cancer cells. Taxol’s mechanism of action involves promoting the formation of stable microtubule bundles and mitotic-like asters, which disrupt normal mitotic spindle function. Taxol efficacy can be influenced by various factors, including the genetic status of the cancer cells [136]. For instance, the expression of certain genes, such as NEK4, can alter the sensitivity of cells to Taxol. Indeed, reducing NEK4 levels in mouse and human lung adenocarcinoma cells appeared to create resistance to Taxol but heightened the sensitivity of the cells to vincristine, which binds tubulin and disrupts microtubule polymerization [134].

The evolving picture of NEK4 in cancer biology emphasizes its potential not only as a single therapeutic target but as an essential molecule in cell cycle regulation (Figure 3; Table 2). Given the roles of NEK4 in disease progression, it has been speculated that this kinase has the potential to be both a therapeutic target and a predictive biomarker for multiple disease states, including lung and colorectal cancers [129]. Taken together, the studies outlined above suggest that inhibiting NEK4 can slow or prevent EMT and metastasis and make existing chemotherapies more effective.

The multifaceted roles of NEK4 present a complex therapeutic landscape. For instance, inhibiting NEK4 could be beneficial in preventing metastasis by blocking EMT; however, the same action could induce resistance to taxane-based chemotherapies, which are a cornerstone of treatment for many cancers. This therapeutic conflict needs to be resolved before NEK4 can be considered a viable drug target. It is essential to understand whether these functions are coupled or can be targeted independently.

### 4.5. NEK5

NEK5 is increasingly being recognized for its role in regulating diverse cellular processes, including centrosome integrity, mitochondrial dynamics, cellular energy homeostasis, and DNA replication stress (Table 2) [9]. Centrosomes, the principal microtubule–organizing centers, orchestrate chromosome segregation during mitosis [137]. Knockdown of NEK5 led to depletion of the pericentriolar material (PCM) components, pericentrin, Cep192, and cyclin-dependent kinase 5 regulatory subunit-associated protein 2 (CDK5RAP2), from centrosomes. This was associated with premature centrosome separation in human osteosarcoma (U2OS) cells. Similar results were observed upon expression of a kinase-dead variant of NEK5. Moreover, siRNA-resistant wild-type, but not the kinase-dead variant, was able to rescue the phenotype. Taken together, this suggests that NEK5-dependent phosphorylation likely contributes to centrosome separation by promoting timely linker disassembly and bipolar spindle assembly. Despite these advances, the precise molecular mechanisms by which NEK5 regulates centrosome dynamics remain unknown. Advanced imaging modalities such as super-resolution microscopy (SRM) promise to elucidate the spatiotemporal interactions of NEK5 with centrosomal scaffolds and their impact on centrosome cohesion and spindle formation [138].

In addition to its role in centrosome dynamics, NEK5 also regulates microtubule assembly and function during mitosis, impacting processes such as cell migration and motility [139]. These functions are particularly relevant in cancer cells, where the regulatory role of NEK5 is altered. For example, during cancer metastasis, NEK5 activity is coordinated with that of other NEK family members, such as NEK4 and NEK6, to orchestrate microtubule organization [140]. This interplay suggests that targeting NEK5 and its associated kinases could provide therapeutic avenues for disrupting aberrant microtubule dynamics in cancer, potentially hindering tumor progression and metastasis [137].

NEK5 also contributes to cellular resilience against stress, positioning it as a key player in metabolic and apoptotic pathways [9]. For instance, NEK5 plays a role in resolving DNA replication stress, especially with respect to DDR pathways [13]. NEK5 interacts with topoisomerase IIβ, facilitating genomic stability by modulating this enzyme critical for DNA replication and repair [9]. Interestingly, in addition to genomic DNA integrity, NEK5 phosphorylates the mitochondrial protease LonP1, which is essential for mtDNA maintenance following oxidative damage (Table 3) [72]. Thus, NEK5 may function as a central regulator of genetic integrity by coordinating DNA stability in both the nucleus and mitochondria.

Importantly, NEK5 is upregulated in various types of cancer, including breast, colon, stomach, lung, and thyroid cancers [118,139,140]. Indeed, the elevated expression of NEK5 correlates with more aggressive tumor phenotypes, such as increased migration and invasion, especially in breast cancer cells. For example, NEK5 promotes a mesenchymal and migratory phenotype in breast cancer, contributing to metastasis [72]. In support of this notion, doxycycline-inducible shRNA systems have demonstrated that NEK5 knockdown affects these traits in breast cancer cell lines, such as MDA-MB-231 and TU-BcX-4IC [139]. The associations of NEK5 with cancer also extend to stomach adenocarcinoma [140]. Bioinformatics tools and databases, such as the cancer cell line encyclopedia (CCLE) and tumor immune estimation resource (TIMER), suggest that NEK5 is differentially expressed in this cancer and could serve as a potential biomarker for prognosis and therapeutic targeting [140,141,142]. However, its effects on cell proliferation and cancer progression appear to be cell type-specific, indicating that the role of NEK5 in cancer can vary depending on the cellular context [139]. Nonetheless, given its involvement in critical cellular processes and its upregulation in cancer, NEK5 represents a promising therapeutic target. Indeed, targeting NEK5 could potentially disrupt cancer cell migration and invasion, offering a strategy to combat metastasis in certain cancers.

### 4.6. NEK6

NEK6 is believed to function primarily during cell cycle regulation, antioxidant defense, and mitotic progression (Figure 3; Table 2). For instance, NEK6 contributes to mitotic spindle assembly and chromosome alignment, both of which are critical for accurate cell division [77]. Specifically, NEK6 kinase activity is vital for centrosome separation and establishing a bipolar spindle, which ensures the proper alignment and segregation of chromosomes during mitosis [1]. This process is coordinated with other kinases, such as cyclin-dependent kinase 1 (CDK1), PLK1, NEK7, and NEK9 to maintain precise control over mitotic events, reflecting a hierarchical regulatory mechanism vital for mitotic events [50]. Moreover, recent studies have revealed interactions between NEK6 and members of the STAT3 signaling pathway, which is a crucial mediator in oncogenesis [143]. For instance, NEK6 has been found to phosphorylate STAT3 on S727 (Table 3) [79]. Interestingly, phosphorylation of this site may impact Janus kinase (JAK)-mediated phosphorylation of the well-known STAT3 regulatory phosphosite at Y705 [144]. Consistent with this notion, Yu and colleagues recently reported that knockdown of NEK6 by the endogenous microRNA, miR-26a-5p, significantly decreased phosphorylation of STAT3 on Y705 [143]. This suggests that NEK6-dependent regulation of STAT3 may play a role in the transcriptional regulation of genes involved in cell proliferation while inhibiting apoptosis-related genes [145].

Consistently, overexpression of NEK6 has also been linked to the etiology and progression of several cancers, including osteosarcoma, breast cancer, prostate cancer, hepatocellular carcinoma, colon cancer, gastric cancer, and ovarian cancer (Figure 3; Table 4). For example, in osteosarcoma, NEK6 accelerates tumor progression through STAT3-mediated signaling, contributing to poor prognosis [146]. In prostate cancer, NEK6 affects redox balance and DDR, influencing the sensitivity of cancer cells to chemotherapeutic agents like cisplatin [147]. Additionally, the role of NEK6 in mitochondrial dynamics and the regulation of cellular antioxidant defenses makes it a potential therapeutic target in castration-resistant prostate cancer (CRPC). 

In ovarian cancer, NEK6 overexpression is associated with adverse outcomes and drug resistance, possibly through its cooperation with HIF-1α [148]. For instance, NEK6 overexpression has been shown to enhance the survival of cancer cells under hypoxic conditions, potentially contributing to tumor progression and metastasis. This is likely mediated by the upregulation of HIF-1α, a critical regulator of the cellular response to low oxygen levels [149]. Studies suggest that NEK6 facilitates the stabilization and transcriptional activity of HIF-1α, leading to increased expression of genes involved in angiogenesis, glycolysis, and cell proliferation [148]. Consequently, targeting the NEK6/HIF-1α axis may represent a novel therapeutic strategy to improve treatment outcomes in patients with treatment-resistant ovarian cancer. The interplay between NEK6 and other signaling pathways involved in cell survival and proliferation suggests that a multifaceted approach may be necessary to effectively inhibit its oncogenic effects [147].

Finally, as alluded to above, NEK6 interaction with microRNAs such as miR-26a-5p, miR-506-3p and miR-323a-3p provides an additional layer to its regulatory role in cancer cell proliferation and apoptosis [143,146]. These miRNAs serve as post-transcriptional regulators of NEK6, modulating its expression and activity, which subsequently impact critical signaling pathways governing cell cycle progression and apoptotic processes [146]. By shifting the balance between cell survival and death, the dynamic interplay between NEK6 and these microRNAs may play a pivotal role in tumorigenesis. Furthermore, the involvement of NEK6 in broader cellular processes such as DDR and redox homeostasis highlights its relevance in diseases driven by genomic instability and oxidative stress [147].

### 4.7. NEK7

In addition to its role in cell cycle regulation and mitosis as part of the NEK6/NEK7/NEK9 signaling axis, NEK7 has also recently gained attention as a regulator of the NLRP3 inflammasome, an essential component of the innate immune system [150]. The NLRP3 inflammasome is a multiprotein complex responsible for activating caspase-1, which processes the pro-inflammatory cytokines, interleukin-1β (IL-1β) and interleukin-18 (IL-18), into their active forms [151,152]. These cytokines are crucial for mediating inflammation, a key defense mechanism against infections and a central driver in the pathogenesis of various inflammatory diseases.

During the innate immune response, potassium efflux triggers the generation of mitochondrially derived ROS (mtROS), which in turn promotes the recruitment of NEK7 to the leucine-rich repeat (LRR) domain of NLRP3 [152,153]. Once there, NEK7 drives a conformational change in NLRP3 that converts it from a closed, inactive state to an open, disk-shaped oligomer, enabling the recruitment of procaspase-1 and the bipartite adaptor protein, apoptosis-associated Speck-like protein containing a CARD (ASC), and leading to caspase-1 activation [154]. Surprisingly, recent studies suggest that, rather than phosphorylating NLRP3, the role of NEK7 in inflammasome activation is entirely structural. Cryo-EM reconstructions and mutational mapping reveal that NEK7 engages LRRs in NLRP3 via a distinct binding epitope, fostering oligomerization without requiring NEK7 kinase activity [155]. Consequently, small molecules and natural products that disrupt this protein–protein interface, such as the NLRP3 inhibitors MCC950, oridonin, and artemisinin, effectively blunt inflammasome assembly and downstream cytokine release in preclinical models [151,156,157]. The ROS-dependent NEK7–NLRP3 interaction underlies inflammatory responses in contexts as diverse as uric acid-induced renal tubular injury and gout [158]. Notably, the specificity of NEK7 for NLRP3 with no recruitment to NOD-like receptor family CARD-containing protein 4 (NLRC4) or absent from melanoma 2 (AIM2) inflammasomes highlights its unique role in linking redox imbalance to innate immunity [152].

Recent studies have extended the immune functions associated with NEK7 to neuroinflammation and other inflammatory disorders. For instance, excessive inflammasome activation contributes to the pathology of gout, atherosclerosis, AD, and systemic lupus erythematosus [159,160,161]. Likewise, in mouse models of traumatic brain injury (TBI), NEK7 is upregulated in injured cortex and hippocampus [157]. Consistently, genetic knockdown or pharmacological inhibition of NEK7 diminishes NLRP3 activation, reduces IL-1β release, and ameliorates neuronal damage and behavioral deficits [157]. These findings position the NEK7–NLRP3 interface as a promising therapeutic target for mitigating secondary inflammation in TBI and other neuroinflammatory disorders.

NEK7 also modulates oxidative stress pathways, tying it to diseases such as cancer and other inflammatory disorders (Figure 3; Table 2 and Table 4). For example, under oxidative stress, NEK7 is recruited to telomeres in an ATM-dependent manner, where it phosphorylates telomeric repeat binding factor 1 (TRF1) on S114 (Table 3) [80]. This modification prevents recognition of TRF1 by the FBX4 E3 ligase, stabilizing the shelterin complex and limiting ROS-induced telomeric attrition and DNA damage signaling. Likewise, in models of acetaminophen-induced acute liver injury, hepatocytic NEK7 expression preserves redox homeostasis by upregulating mitochondrial superoxide dismutase (SOD2), maintaining glutathione pools, decreasing lipid peroxidation, and sustaining ATP production [86]. Loss of NEK7 in this context leads to exacerbated ROS accumulation and cell death, whereas NEK7 overexpression confers hepatoprotection [156]. By safeguarding telomere integrity and amplifying antioxidant defenses, NEK7 supports genomic stability and cell survival under oxidative challenge. Dysregulation of these redox-related functions can promote telomere dysfunction and genomic instability in cancer.

Together, these findings suggest that NEK7 is a multifaceted node integrating mitotic control, redox sensing, genome maintenance, and immune activation, emphasizing its broad significance in both physiological and pathological settings. However, several critical questions still remain to be answered. For instance, the discovery of a non-catalytic, purely structural role for NEK7 in inflammasome assembly is a fascinating example of protein evolution. However, why a kinase was co-opted for this function is unclear. It would be useful to elucidate more information on the evolutionary advantage of using a kinase as a scaffold. Furthermore, while potassium efflux and ROS are known triggers for the NEK7-NLRP3 interaction, the precise signaling events that regulate the availability and localization of NEK7 for this specific function are still poorly defined and represent a critical area for future research. Finally, while NEK6 and NEK7 share 85% sequence identity in their catalytic domains and are often discussed as a functional pair activated by NEK9, a key unanswered question is the degree of their functional redundancy. Do they have distinct, non-overlapping substrates and roles, or do they act interchangeably to ensure robust mitotic progression? Differentiating their specific functions is critical for drug development, as targeting a single kinase may be ineffective if its paralog can fully compensate.

### 4.8. NEK8

NEK8 is involved in the regulation of several cellular signaling pathways, though the precise mechanisms are not well understood. For instance, NEK8 is known to interact with the small GTPase, Ras-related nuclear protein (RAN), which is crucial for nuclear-cytoplasmic transport [40]. RAN, which catalyzes the hydrolysis of the γ-phosphate of guanosine triphosphate (GTP) to produce guanosine diphosphate (GDP), is essential for several cellular processes, particularly those related to nuclear transport, mitotic spindle assembly, and nuclear envelope assembly [162,163].

NEK8 has also been implicated in the Hippo signaling pathway through its interaction with Yes-associated protein (YAP), a transcriptional co-activator critical for controlling cell proliferation and organ size [164]. Specifically, NEK8 is believed to influence organ size by balancing cell proliferation and cell cycle arrest. It also affects the phosphorylation state and nuclear shuttling of other transcriptional co-factors involved in the Hippo signaling pathway, such as transcriptional co-activator with PDZ-binding motif (TAZ), favoring TAZ’s nuclear translocation [164].

NEK8 also influences several key cellular processes. For instance, NEK8 provides a protective function in cells experiencing endoplasmic reticulum (ER) stress, potentially delaying apoptosis through interaction with the molecular chaperone, heat shock protein 70 (HSP70) [165]. Likewise, although NEK8 is not a primary regulator of the cell cycle, its interactions with other members of the NEK family suggest some involvement in this process [1,70].

The role of NEK8 in ciliogenesis, which entails the formation of cilia important for cellular signaling and movement, is especially significant in PKD and related ciliopathies (Figure 3; Table 2 and Table 4). For instance, in PKD, mutations in NEK8 have been linked to ciliary defects that lead to aberrant activation of pathways, such as mammalian target of rapamycin (mTOR) and Wnt, that contribute to cyst formation and growth [40]. Recent studies have also highlighted the impact that NEK8 mutations have on other renal pathologies, such as nephronophthisis, which is characterized by fibrosis and cysts in the kidneys [166,167]. These mutations impair NEK8 kinase activity and its ability to regulate ciliary function, leading to disrupted kidney architecture. These studies also demonstrated that alterations in NEK8 can lead to renal cystic dysplasia, causing severe developmental abnormalities by dysregulating key signaling pathways, like YAP-dependent cell cycle progression [168]. Moreover, due to the widespread presence of primary cilia in various tissues, NEK8-related ciliopathies can affect multiple organ systems, leading to complex syndromes that include liver fibrosis, retinal degeneration, and skeletal abnormalities [40]. The role of NEK8 in regulating cilia formation and function suggests that targeting these pathways could mitigate disease progression. Thus, therapies aimed at restoring NEK8 activity or compensating for its dysfunction could be transformative in managing ciliopathies.

Previous research has also explored the broader implications of NEK8 mutations beyond the ciliopathies. For instance, overexpression of NEK8 in several cancer cell lines, including U2OS osteosarcoma, HepG2 hepatocarcinoma, Sk-Hep-1 endothelial, and MCF-7 breast cancer cells, suggests a role in tumorigenesis, where its involvement in replication stress response and DNA repair pathways may aid cancer cell survival and proliferation [165,169,170,171]. In this context, the engagement of NEK8 with the Hippo pathway is noteworthy, as it interacts with YAP and TAZ to promote their nuclear localization and drive transcription of proliferative targets [164]. NEK8 also serves as a scaffold for components of the ATR–CHK1 axis at stalled replication forks, facilitating CHK1 activation, suppressing cyclin-A–CDK activity and stabilizing RAD51 filaments to maintain fork integrity during S phase [165].

### 4.9. NEK9

Though NEK9 is involved in primary cilia formation by acting as an autophagy adaptor for myosin heavy chain 9 non-muscle isoform IIA (MYH9IIA), a growing body of evidence suggests that it is also involved in several cellular processes related to cancer (Table 4) [172]. For instance, NEK9 is an essential regulator of cell division and plays a key role in spindle organization, cell cycle progression, and DDR (Table 2) [1,173]. NEK9 has been implicated in the maintenance of genomic integrity during replicative stress. Specifically, NEK9 formed a complex with CHK1 to aid in the Gemcitabine-induced DNA damage response in triple negative breast cancer cells [173]. Consistently, siRNA mediated depletion of NEK9 resulted in accumulation of early DNA damage marker, γH2AX, and the DNA repair complex, replication protein A (RPA70), in U2OS osteosarcoma cells. Interestingly, NEK9 has also been shown to undergo autophosphorylation and nuclear localization during interphase by forming a complex with the histone chaperone complex, facilitates chromatin transcription (FACT), suggesting that NEK9 may play a role during transcriptional elongation [174].

NEK9 also acts as an upstream activator of NEK6 and NEK7 during prophase and prometaphase, orchestrating a mitotic kinase cascade necessary for centrosome separation and spindle assembly (Table 3) [74,86]. NEK9 activation, in turn, is tightly regulated by phosphorylation events mediated by CDK1 and PLK1 during mitosis, which relieves an autoinhibitory state and enables NEK9 to achieve full catalytic activity [55]. Once activated, NEK9 localizes to centrosomes and spindle poles during the early stages of cell division, where it is believed to aid in the recruitment of γ-tubulin to MTOCs [175]. This recruitment is crucial for the nucleation and organization of spindle microtubules. Consistent with a role for NEK9 in this process, studies suggest that depletion of NEK9 impairs spindle assembly and leads to defects in chromosome segregation, as evidenced by the dispersion of γ-tubulin from spindle poles and the activation of the SAC [1].

Likewise, NEK9-mediated activation of NEK6 and NEK7 led to phosphorylation of the kinesin, Eg5, on S1033. Eg5 phosphorylation on this site promotes centrosome separation and timely mitotic progression, which is fundamental for the prevention of aneuploidy [43,44]. NEK9 also influenced mitotic processes through its phosphorylation of NEDD1, a microtubule nucleation adaptor protein, and by activating NEK6 and NEK7, as outlined above (Table 3) [5,43,74,86,87,175,176].

In addition to its role in cell division, NEK9 is also involved in the regulation of cell motility and metastasis. For instance, Lu et al. found that NEK9 is associated with roundabout guidance receptor 1 (ROBO1) and the axon guidance protein, Slit guidance ligand 2 (SLIT2), in the gastric cancer cell lines, AGS and MKN45 [88]. Formation of the NEK9-ROBO1-SLIT2 ternary complex promoted NEK9-mediated phosphorylation of the transcriptional elongation factors, tripartite motif containing 28 (TRIM28) and cortactin (CTTN), which helped to promote metastasis in gastric cancer cells (Table 3) [88]. Consistently, NEK9 expression is significantly higher in gastric cancer compared to their noncancerous counterparts, as shown by immunohistochemistry (IHC) analysis [89]. Upregulation of NEK9 expression is also correlated with advanced tumor stages and reduced overall survival, indicating that NEK9 might serve as a prognostic marker in various cancers. Furthermore, high NEK9 levels are linked to lymph node and distant metastases, underscoring its potential role in metastasis. In this context, NEK9 functions as an effector of the interleukin-6 (IL-6)/STAT3 signaling pathway, which contributes to cancer metastasis. For example, Lu et al. recently explored the role of NEK9 in the IL-6 pathway during gastric cancer metastasis [89]. These studies suggest that NEK9 regulates metastasis by promoting the phosphorylation of Rho/Rac guanine nucleotide exchange factor 2 (ARHGEF2), which is a GEF that plays a critical role in cell motility and cytoskeletal reorganization. ARHGEF2 activates RhoA, a small GTPase involved in the regulation of the actin cytoskeleton and cell movement (Table 3) [89]. The ability of NEK9 to phosphorylate ARHGEF2 enhances its activity, leading to increased cell motility and metastatic potential in gastric cancer cells. This phosphorylation event is a key step in the NEK9-mediated metastasis pathway.

NEK9 influences cell movement through its interaction with the NEK7 pathway [78]. Specifically, NEK9-dependent activation of NEK7 has been linked to changes in cell morphology and increased migration in interphase cells [1]. Likewise, activation of NEK9 (and NEK7) promoted the activation of oncogenic EML4–ALKv3 and V5 fusion proteins that are involved in the progression of certain cancers, including non-small cell lung cancer (NSCLC) and gastric cancer (Figure 3; Table 4) [177]. Once activated, EML4-ALKv3 contributes to disease progression by stabilizing microtubules and enhancing the rate of migration of cancer cells. In NSCLC, NEK9 expression is associated with EML4–ALK fusion variants, particularly V3 and V5, that has been shown to correlate with poor overall survival in patients [177].

Together, these findings suggest that NEK9 may be an attractive therapeutic target for slowing or even halting the spread of certain cancers harboring EML4–ALK fusions, especially gastric cancer or NSCLC. Combining targeted NEK9 treatments with existing therapies like ALK inhibitors or microtubule inhibitors could improve patient outcomes [118]. Furthermore, NEK9 inhibitors have shown potential in halting metastatic processes in gastric cancer [89]. Given its critical roles in cell division and potential involvement in cancer progression, NEK9 represents an attractive target for drug development, offering therapeutic benefits by disrupting mitotic spindle formation and potentially inhibiting cancer cell migration and metastasis [44].

### 4.10. NEK10

NEK10, which is the only dual specificity kinase in the NEK family [16], plays crucial roles in diverse cellular processes, including mitochondrial dynamics and protein homeostasis (Figure 3; Table 2). For instance, stable knockdown of NEK10 in HeLa cells led to several mitochondrial abnormalities, including alterations in mitochondrial morphology, mitochondrial oxygen consumption, and mitochondrial DNA integrity. For instance, de Oliviera et al. recently found that NEK10 KO in human HAP1 chronic myelogenous leukemia cells affected mitochondrial homeostasis as well as the phosphorylation levels of respiratory complexes and other factors critical for mitochondrial function [178]. NEK10 KO resulted in a marked increase in the number of spherical/fragmented mitochondria in HAP1 cells (increasing from ~15% in control cells to >40% in NEK10 KO cells). The increased fragmentation correlated with a 2–3-fold decrease in mitochondrial respiration in NEK10 KO cells. Phosphoproteomics analysis using isolated mitochondria from parental and NEK10 KO cells revealed that the phosphorylation status of several factors critical for mitochondrial function, including NADH:ubiquinone oxidoreductase subunit B4 (NDUFB4; a component of Complex I) and translocase of the outer mitochondrial membrane 20 (TOM20), were decreased more than 2-fold following NEK10 KO [179]. Together, these studies suggest that NEK10 may regulate mitochondrial homeostasis and function.

NEK10 is also involved in the regulation of several other cellular processes. For instance, it has been hypothesized that NEK10 helps to ensure that proteins are correctly folded, modified, and degraded, thereby maintaining cellular health and functionality [118]. Consistent with this notion, recent studies have highlighted the involvement of NEK10 in the unfolded protein response (UPR), a mechanism activated under stress conditions to restore normal protein folding in the ER. Indeed, NEK10 activity is necessary for preventing the accumulation of misfolded proteins, which can lead to cellular dysfunction and disease [178]. Moreover, NEK10 interactions with β-catenin through its ARM motifs further position it as a multifunctional kinase regulating cellular energy and genomic stability [54]. NEK10 also supports genomic stability by facilitating RAD51 recruitment to DNA damage foci, thereby enhancing homologous recombination repair and protecting against oncogenic transformation [9].

NEK10 is also known to participate in cell cycle regulation and DDR [1]. In this context, NEK10-mediated phosphorylation of the tumor suppressor, p53, on Y327 is associated with cell cycle arrest, linking NEK10 to critical DDR pathways [49]. However, it is not currently clear how Tyr phosphorylation competes with (or complements) Ser/Thr phosphorylation events that are known to regulate p53 activity. Likewise, NEK10 has been shown to act as a key mediator of the G2/M DNA damage checkpoint following UV irradiation by selectively engaging the ERK1/2 cascade [9]. Upon UV exposure, NEK10 assembled into a ternary complex with RAF1 and MEK1. However, rather than altering the intrinsic kinase activity of RAF1, NEK10 promoted MEK1 autoactivation, which in turn drove ERK1/2 phosphorylation. Activated ERK1/2 then enforced G2/M arrest by stabilizing checkpoint effectors and preventing mitotic entry until DNA lesions were repaired, a mechanism that appears to be uniquely responsive to genotoxic stress rather than to mitogenic stimuli [9]. This UV-specific regulation underscores the pivotal role that NEK10 plays in preserving genomic integrity under conditions of DNA damage.

In the context of Wnt signaling, NEK10 uniquely functions as a dual-specificity kinase by phosphorylating β-catenin on Y30, thereby modulating the proteolytic turnover of the transcription factor [54]. Association of NEK10 with the Axin–CK1–GSK3 destruction complex facilitated this Tyr modification, which is a prerequisite for subsequent GSK3-mediated Ser phosphorylation and ubiquitin-dependent degradation. Indeed, loss of Y30 phosphorylation impaired GSK3 targeting, leading to β-catenin stabilization and nuclear accumulation, ultimately driving Wnt target gene expression [54]. Through this precise phosphorylation event, NEK10 appears to fine-tune β-catenin levels to influence cellular proliferation and fate decisions.

Given its diverse roles and regulatory complexity, NEK10 presents an intriguing target for drug development. Therapies aimed at modulating NEK10 activity could potentially enhance protein homeostasis, offering benefits in treating diseases characterized by protein misfolding and aggregation, such as neurodegenerative disorders (Figure 3; Table 4). For instance, the ability of NEK10 to modulate the UPR could provide therapeutic benefits by enhancing cellular resilience to stress [178]. Additionally, targeting NEK10 in cancer could disrupt oncogenic pathways and suppress tumor growth, providing a novel avenue for cancer therapy. Indeed, NEK10 regulatory functions might influence tumor progression by affecting the stability and activity of oncogenic proteins, making it a potential target for therapeutic intervention [54].

### 4.11. NEK11

Recently, NEK11 has emerged as a player in cellular stress responses and genome stability. Its role in DDR is particularly noteworthy, as NEK11 regulates the G2/M cell cycle checkpoint (Figure 3; Table 2). This regulation ensures that cells do not proceed to mitosis with damaged DNA, thereby maintaining genomic integrity and preventing the propagation of mutations that could lead to cancer [91]. NEK11 achieves this by phosphorylating the CDC25A phosphatase on several residues in its N-terminus, including S82, marking it for degradation and arresting the cell cycle until DNA is repaired (Table 3). By controlling CDC25A degradation in response to DNA damage, NEK11 ensures proper cell cycle progression and prevents tumorigenesis. NEK11 also impacts the G2/M checkpoint by regulating chromatin accessibility during DDR.

Beyond its role in DDR, NEK11 is also involved in various cellular functions, supporting its functional diversity. For example, NEK11 plays a role in centrosome duplication and mitotic spindle assembly, both critical for accurate cell division [180]. The kinase activity of NEK11 is tightly regulated by upstream signals, including phosphorylation events mediated by other kinases like CHK1, which modulates its activity in response to cellular requirements [92]. This regulation allows NEK11 to integrate signals from the DDR pathway, coordinating cellular responses and ensuring proper cell cycle progression.

The involvement of NEK11 with other kinases also highlights its role in a complex signaling network. For instance, its interaction with CHK1 enhances NEK11 activity, ensuring a robust response to DNA damage [92]. This crosstalk with other cell cycle regulators ensures that NEK11-regulated functions align with the overall cellular response to stress and damage. Its role as a gatekeeper of genomic integrity underscores its therapeutic potential in cancer prevention [181]. Indeed, modulating NEK11 activity could enhance the efficacy of cancer treatments, such as chemotherapy and radiation therapy, by hindering DDR pathways and promoting cancer cell apoptosis. Furthermore, targeting NEK11 could help overcome drug resistance in cancers where resistance to genotoxic chemotherapeutic agents is a challenge by preventing the repair of DNA damage induced by treatments. As research progresses, further insights into the diverse roles of NEK11 and its regulatory mechanisms are expected to reveal additional therapeutic opportunities.

## 5. Perspectives and Future Directions

The NEK family of kinases encompasses a diverse array of functions crucial to cellular homeostasis. However, significant gaps remain in our understanding of their biological roles and therapeutic potential. Future investigations into specific NEK family members will be pivotal in unlocking new insights and advancing their application in disease contexts.

NEK1, for example, warrants further exploration of its broader implications in neurodegenerative diseases beyond ALS and PD. Particular attention should be given to its role in modulating mitochondrial function and neuroinflammatory pathways that are critical to neuronal survival and function. Similarly, NEK2 holds promise as a therapeutic target in several cancers, with a need for deeper investigation into its contributions to drug resistance and chromosomal instability in aggressive tumor phenotypes.

Meanwhile, the regulatory influence of NEK3 on estrogen receptor signaling and its pivotal role in cell motility during cancer metastasis also present exciting opportunities for further study, especially regarding its interactions with microtubules. NEK4 involvement in EMT and mitochondrial fission highlights another area ripe for exploration, particularly its potential to enhance the efficacy of chemotherapeutic agents like Taxol. NEK5 presents an intriguing avenue for research into microtubule stability and dynamics, both during the cell cycle and in non-dividing cells, with implications for intracellular transport, cell signaling, and structural integrity. In the future, it will be interesting to explore the role that NEK5 plays in microtubule stability and dynamics throughout cell cycle and in non-dividing cells. This includes its impact on intracellular transport, cell signaling, and structural integrity in neuronal cells where microtubule dynamics are critical [111].

NEK6 and NEK7 stand out from other NEK family members due to their relatively simple structure. Unlike NEK2, which includes both a catalytic domain and a regulatory C-terminal region, NEK6 and NEK7 are primarily composed of the catalytic domain alone. This streamlined structure suggests that NEK6 and NEK7 might have overlapping roles, with NEK9 likely acting as their upstream activator, phosphorylating them in a sequential manner [154]. Interestingly, the simpler architecture of NEK6 and NEK7 may reflect an evolutionary adaptation, enabling them to quickly engage in specific signaling pathways without the need for elaborate regulatory mechanisms. This structural and functional simplicity could reflect an evolutionary adaptation, allowing NEK6 and NEK7 to efficiently participate in specific signaling pathways without the need for complex regulatory mechanisms.

NEK6 and NEK7 also represent critical players in mitotic spindle assembly, yet their roles extend to immune signaling, notably NEK7 function in inflammasome activation. A deeper understanding of the interplay between these kinases could illuminate new therapeutic opportunities. The regulation of NEK6 is pivotal in disease contexts, as genetic variations can disrupt its catalytic activity and impact cellular processes, including cell cycle checkpoints and DNA repair [182]. Targeting NEK6 could offer therapeutic potential by disrupting oncogenic pathways like STAT3 signaling, thereby reducing cancer cell proliferation and enhancing apoptosis [146]. Combining NEK6 inhibitors with chemotherapeutic agents might improve treatment efficacy, particularly in cancers where NEK6 is overexpressed, such as breast cancer. Moreover, monitoring NEK6 levels and its regulatory interactions could provide valuable insights into cancer diagnosis and prognosis [182].

NEK8, on the other hand, is closely linked to ciliogenesis and kidney disease pathophysiology, particularly in ciliopathies, where its contributions to cellular signaling and structural maintenance remain underexplored. The role of NEK9 in metastasis and its interaction with the NEK6/NEK7 axis in regulating microtubules also merits further investigation, as does NEK10 involvement in the UPR and mitochondrial homeostasis. Lastly, NEK11-dependent regulation of DDR and its capacity to maintain genomic stability are key areas of interest, with significant implications for cancer therapy.

Expanding our knowledge of the NEKs will not only advance our understanding of the fundamental roles of this kinase family in cell biology but it will also underscore their translational potential in developing therapeutic strategies across a broad spectrum of diseases. For instance, as the field advances, a more complete understanding of NEK biology promises to illuminate fundamental principles of cell cycle regulation, genome maintenance, and organelle dynamics. At the same time, it could unlock new avenues for therapeutic intervention across a broad spectrum of diseases. By bridging the current knowledge gaps, future work has the potential to transform the NEKs from an understudied kinase family into a fertile source of insights and drug targets with far-reaching biomedical implications, as discussed below.

### Future Directions

Although substantial progress has been made in elucidating the biological importance of NEK family members, fully mapping their substrate repertoires remains an urgent priority. Functional screening assays such as OPLS have defined consensus motifs for individual NEKs, yet many physiological targets remain elusive (particularly those operating outside mitosis). Combining proximity-labeling techniques (e.g., BioID or TurboID) with unbiased phosphoproteomics during discrete cell-cycle phases or stress conditions should reveal both stable and transient NEK interactors [183,184]. Pairing these datasets with genome-wide CRISPR LoF screens would help prioritize the most functionally relevant substrates for mechanistic validation. Likewise, the identification of kinase-substrate relationships using functional protein microarrays could offer new insights into the biochemical targets of NEK family members [185,186,187].

A deeper understanding of NEK conformational dynamics will likewise be critical. To date, structural studies have captured only isolated domains or autoinhibitory “Tyr-down” states (as in NEK7), leaving full-length kinase–substrate complexes largely uncharted. Cryo-EM studies of near-full-length NEKs bound to native partners such as NEK9 in complex with NEK6/7 or NEK1 with C21orf2 combined with time-resolved HDX-MS, can provide critical insights into the structural transitions underpinning activation, substrate recognition, and allosteric regulation [188]. Integrating these experimental insights with predictive modeling (for example, AlphaFold 3 or AlphaFold-Multimer) could accelerate the rational design of allosteric inhibitors that exploit dynamic pockets unique to each NEK [189].

At the same time, the development of selective small-molecule inhibitors for NEKs will be important both for therapeutic applications and for pharmacological dissection of NEK function. However, this task remains challenging, due in large part to the high structural similarity among family members in their active sites, which can lead to off-target effects, and the dynamic conformational behavior of kinases, which complicates the design of molecules that effectively stabilize active or inactive states. The NEKs current position in the “dark kinome”, which includes kinases with limited functional and structural characterization, further hampers the identification of unique binding sites and the rational design of inhibitors. Despite these hurdles, several small-molecule inhibitors targeting NEK family members have been reported. For instance, NEK1 can be inhibited by BSc-5367 while NEK2 has multiple inhibitors, including the ATP-competitive inhibitor, CMP3a, the allosteric inhibitor, NCL-00016066, the irreversible covalent inhibitor JH295, and the indirect inhibitor INH1, which disrupts NEK2–Hec1 interactions [190]. However, the majority of the NEK family currently lack selective small-molecule inhibitors, underscoring the need for continued medicinal chemistry efforts.

The development of tools to track changes in NEK activity with subcellular precision, such as genetically encoded fluorescent biosensors, will also be transformative [191,192,193]. Many NEK family members operate within restricted locales. For example, NEK1 often acts at DNA damage foci whereas NEK7 and NEK8 function at the NLRP3 inflammasome and at the ciliary base, respectively [155]. Yet existing tools often lack the spatiotemporal resolution to monitor dynamic changes in the activity profiles of these NEK pools. Similarly, future tool development efforts could focus on organelle-targeted optogenetic switches and conditional degron systems that enable dynamic control of individual NEKs in living cells [194,195]. In parallel, chemical biology strategies to develop PROTACs or highly selective allosteric modulators will enable clean functional dissection both in situ and in vivo [196].

Translating NEK biology into disease models is another vital frontier. Although dysregulation of NEKs is implicated in cancers, ciliopathies, and neurodegeneration, there is a shortage of genetically accurate models carrying patient-derived mutations (for instance, ALS-linked NEK1 variants). Generating CRISPR knock-in organoids such as kidney or neuronal models and conditional mouse alleles will more faithfully recapitulate human pathophysiology and allow longitudinal multi-omics profiling under genotoxic or metabolic stress. These platforms will be invaluable for validating small-molecule inhibitors or degraders as precision medicine interventions.

Finally, embracing systems-level and computational approaches will uncover emergent properties of NEK networks that are not apparent from single-kinase studies. By integrating phosphoproteomic maps, protein–protein interaction networks, and single-cell imaging data into quantitative models, researchers can identify feedback loops, compensatory pathways, and novel intervention points. Machine-learning algorithms applied to these multi-dimensional datasets may predict synergistic drug combinations such as co-inhibition of NEK2 and PLK1 in aggressive tumors and streamline the path from discovery to therapy.

The convergence of advanced structural techniques, spatially resolved chemical and genetic tools, genetically tractable disease models, and systems-level modeling promises to accelerate NEK research. By addressing these future directions, the field is poised to unlock fundamental insights into the roles of NEKs in cell-cycle control, ciliary biology, DNA damage responses, and innate immunity and to translate those insights into targeted therapies.

## 6. Conclusions

The NEK family of S/T kinases has evolved from a single ancestral NIMA-like kinase into an eleven-member branch of the human kinome with striking structural diversity and functional breadth. These kinases collectively orchestrate fundamental cellular processes, including centrosome disjunction, ciliary assembly, microtubule organization, DNA damage sensing and repair, cell-cycle checkpoint control, and inflammatory signaling. Their architecture reflects this complexity: while all NEKs share a conserved catalytic core, the acquisition of diverse accessory domains—including coiled-coil motifs, PEST sequences, DEAD-box helicase-like modules, RCC1-like repeats, and armadillo domains—has enabled lineage-specific innovations in regulation, substrate selection, and subcellular targeting. These structural features underlie their finely tuned activation mechanisms, involving multisite phosphorylation, dimerization, and scaffold-mediated conformational switching, which allow each NEK to execute context-specific signaling roles.

This functional diversification has profound implications for human health. Nearly all NEK family members have been implicated in human diseases, with aberrant NEK signaling contributing to cancer, ciliopathies, neurodegenerative disorders, and chronic inflammatory syndromes. In cancers, NEKs such as NEK1, NEK2, NEK6, and NEK11 promote genome instability and tumor progression, while others, like NEK1, paradoxically offer opportunities for radiosensitization. Inherited or acquired dysfunction of NEKs also disrupts ciliary and centrosomal homeostasis, as seen in PKD and Joubert syndrome, and compromises neuronal viability, as exemplified by ALS- and PD-associated NEK1 variants. These pathophysiological links emphasize that NEKs are not isolated mitotic kinases but central integrators of cellular architecture, genome integrity, and stress response networks. However, significant gaps remain in our understanding of their biological roles and therapeutic potential. Future investigations into specific NEK family members will be pivotal in unlocking new insights and advancing their application in disease contexts.

## Figures and Tables

**Table 1:** Functional Domains of NEK family kinases. The functional domains in NEK family kinases contribute to their specific cellular functions, including roles in protein degradation (PEST sequence), RNA processing (DEAD-box domain), scaffolding and interaction (RCC1-like domain, Armadillo repeat domain), oligomerization and targeting (coiled-coil domain), and enzymatic activity (catalytic domain).

Domain	NEK Family Members	Location	Functional Role
PEST sequence	NIMA; Fin1 (*S. pombe*); 5 of 11 human NEKs (e.g., NEK2A, 9, 10, 11)	Generally in the C-terminal non- catalytic region	Signals for rapid degradation by ubiquitin-proteosome system; ensures timely turn-over at mitotic exit to coordinate cell cycle progression
DEAD-box domain	NEK5	C-terminal to the kinase domain	ATP-dependent RNA helicase activity; remodels RNA/RNPs; likely links NEK signaling to RNA processing, stress granule dynamics, or mitochondrial homeostasis
RCC1-like domain	NEK8, NEK9	C-terminal to the kinase domain	Β-propeller scaffold for RAN-GTP interactions; enforces autoinhibition (NEK9), scaffolds NEK6/7 activation cascade; may target NEKs to chromatin or centrosomes
Coiled-coil domain	All human NEKs except NEK4, 6, 7; NIMA	C-terminal tail or flanking kinase domain	Drives homo- or hetero-oligomerization; enables trans-autophosphorylation (e.g., NEK2 dimerization); governs subcellular targeting (e.g., centrosome, nucleolus)
Armadillo Repeat domain	NEK10	N-terminal region (4 tandem repeats)	Provides an elongated scaffold for protein–protein interactions; directs NEK10 to ciliary or stress-response complexes and mediates interactions (e.g., with HSPB1)
Kinase domain	All NEK family members	N-terminal 250–300 aa bilobed kinase fold	Binds ATP and catalyzes the transfer of phosphate to protein substrates; controlled by motifs (e.g., Gly-rich loop, VAIK, HRDLKPEN, DFG, and APE motifs) and regulatory features (“Tyr-down” autoinhibition, dimerization cues)

**Table 2:** Involvement of NEK Family Members in Select Cellular Processes. Summary of the involvement of human NEK family members (NEK1–NEK11) across key cellular processes. Green reflects strong, mechanistically supported involvement while yellow represents indirect or limited involvement and red represents no reported role.

Function	NEK1	NEK2	NEK3	NEK4	NEK5	NEK6	NEK7	NEK8	NEK9	NEK10	NEK11
DNA Damage Response											
Cell Cycle Regulation											
Ciliary Function											
Inflammation/ Immune Response											
Microtubule Dynamics/ Intracellular Transport											
Metabolism/ Mitochondrial Function											
Cell Migration											
Apoptosis											
Hypoxia/ Redox Homeostasis											
Unfolded Protein Response											

**Table 3:** Summary of Select NEK–Substrate Relationships. Overview of select kinase-substrate relationships (KSRs) for the NEK family kinases (NEK1–NEK11). The entries are organized according to the NEK family member, the identified substrate, phosphorylation site(s) when known, the functional consequence of the phosphorylation event, and the supporting literature references. Data were compiled from curation of the literature. Functions span key processes such as mitotic progression, centrosome separation, DNA damage response, ciliary signaling, mitochondrial metabolism, and innate immunity. Phosphorylation site specificity is included where experimentally validated.

NEK Member	Substrate	Phosphosite	Function of Phosphorylation	Ref
NEK1				
	ATRIP	T1989	Interacts with ATR to maintain genomic stability in DNA damage repair pathways.	[59]
	Rad54	S572	Promotes the removal of Rad51 from chromatin during homologous recombination (HR) in the G2 phase of the cell cycle	[61]
	C21ORF2	ND	Inhibits ubiquitylation, stabilizing C21ORF2	[62,63]
NEK2				
	c-NAP1	S2131/T2132, S2128, S2229, S2234, S2322	Separation of duplicated centrosomes at the onset of mitosis	[64]
	Rootletin	NH2- and COOH-term fragments	Triggers centrosome separation during the cell cycle	[65]
	HEC1	S165	Enhances HEC1 interaction with MAD1 at kinetochores	[66]
	NLP	ND	Triggers NLP’s removal from the centrosome, allowing reorganization of the microtubule network required for spindle formation.	[67]
	Centrobin	T35, S36, S41, S45	Antagonizes centrobin’s microtubule-stabilizing activity	[68]
	β-catenin	S33, S37, T41	Stabilizes β-catenin	[69]
NEK3				
	VAV-2	ND	Activates VAV2 during prolactin receptor signaling, leading to cytoskeletal reorganization and motility in breast cancer cells.	[70]
NEK4				
	yH2AX	S139 (predicted)	Essential for the formation of γH2AX foci, a key marker of DSBs.	[71]
NEK5				
	LonP1	ND	Modulates LonP1 activity and affects mitochondrial function	[72]
NEK6				
	EML4	S144	Reduces its affinity for microtubules, promoting chromosome congression during mitosis	[73]
	Eg5/KIF11	S1033	Regulation of spindle dynamics	[74]
	Histone H1	ND	Regulation of chromatin condensation during mitosis	[75]
	Histone H3	ND	Regulation of chromatin condensation during mitosis	[75]
	HSP72/HSPA1A	T66	Regulation of spindle organization	[76]
	β-tubulin	T166	Promotes the depolymerization of cortical microtubules (CMTs)	[77]
	p70S6K	T412	Activation of enzymatic activity	[78]
	STAT3	S727	Promotes tumor growth	[79]
NEK7				
	EML4	S146	Modulates microtubule stability	[73]
	TRF1	S114	Stabilizes the shelterin complex and limits ROS-induced telomeric attrition and DNA damage signaling.	[80]
	NLRP3	S803	NEK7 binds to and activates NLRP3 post-K^+^ efflux (non-catalytic); phosphorylation-independent interaction	[81]
	p70S6K	T412	Activation of enzymatic activity	[78]
	Eg5/KIF11	S146, S1033	Regulation of spindle dynamics	[82]
NEK8				
	ANKS6	ND	Localization to the ciliary inversin compartment (IC)	[83]
	MYC	S405	Inhibits ubiquitylation, stabilizing c-MYC	[84]
	BICD2	ND	Potential role in modulating microtubule morphology	[85]
	NEK8 (Autophos)	ND	NEK8 functionally depends on CEP164 for localization and interaction; may act downstream	[83,84]
NEK9				
	NEK6	S206	Activates NEK6, which is crucial for proper spindle formation	[74]
	NEK7	S195	Activates NEK7, which regulates centrosome separation	[86]
	NEDD1	S377	NEDD1 recruitment to centrosomes and proper γ-tubulin localization in mitotic cells	[87]
	TRIM28	S473	Enhances the transcriptional activity, promoting downstream expression of STAT3, NF-κB p100, and cortactin (CTTN), driving cytoskeletal reorganization and metastatic behavior in gastric cancer cells	[88]
	CTTN	S417	Facilitates actin cytoskeletal remodeling and cell motility in the context of gastric cancer metastasis	[88]
	ARHGEF2	ND	Activates ARHGEF2, promoting RhoA activation and cell motility	[89]
	BICD2	ND	Regulates dynein–dynactin motor activity during mitosis	[90]
NEK10				
	p53	Y327	Modulates p53’s stability and activity	[49]
	β-catenin (CTNNB1)	Y30	Promotes degradation of CTNNB1	[54]
NEK11				
	CDC25A	S79, S82, S88	Promotes CDC25A’s degradation in response to DNA damage	[91,92]
	CHK1	S273	Part of the DDR pathway, specifically regulating the G2/M checkpoint	[92]

**Table 4:** Involvement of NEK Family Members in Human Diseases. Summary of NEK family members (NEK1-NEK11) involved in various human diseases. Green cells indicate clear involvement, yellow cells indicate potential or limited involvement, and red cells indicate no known involvement. The diseases assessed include cancer such as breast cancer, ciliopathies such as polycystic kidney disease (PKD), neurodegenerative disorders such as Parkinson’s disease (PD), and inflammatory disorders.

Disease	NEK1	NEK2	NEK3	NEK4	NEK5	NEK6	NEK7	NEK8	NEK9	NEK10	NEK11
Cancer											
Ciliopathies (e.g., PKD)											
Neurodegenerative Disorders											
Inflammatory Disorders											

## Data Availability

Not applicable.

## References

[1] Fry A.M., O’Regan L., Sabir S.R., Bayliss R. (2012). Cell cycle regulation by the NEK family of protein kinases. J. Cell Sci..

[2] Osmani A.H., McGuire S.L., Osmani S.A. (1991). Parallel activation of the NIMA and p34cdc2 cell cycle-regulated protein kinases is required to initiate mitosis in *A. nidulans*. Cell.

[3] Moniz L.S., Dutt P., Haider N., Stambolic V. (2011). Nek family of kinases in cell cycle, checkpoint control and cancer. Cell Div..

[4] Fry A.M., Meraldi P., Nigg E.A. (1998). A centrosomal function for the human Nek2 protein kinase, a member of the NIMA family of cell cycle regulators. EMBO J..

[5] Belham C., Roig J., Caldwell J.A., Aoyama Y., Kemp B.E., Comb M., Avruch J. (2003). A mitotic cascade of NIMA family kinases. Nercc1/Nek9 activates the Nek6 and Nek7 kinases. J. Biol. Chem..

[6] Moniz L.S., Stambolic V. (2011). Nek10 mediates G2/M cell cycle arrest and MEK autoactivation in response to UV irradiation. Mol. Cell. Biol..

[7] Fry A.M., Schultz S.J., Bartek J., Nigg E.A. (1995). Substrate specificity and cell cycle regulation of the Nek2 protein kinase, a potential human homolog of the mitotic regulator NIMA of *Aspergillus nidulans*. J. Biol. Chem..

[8] Parker J.D.K., Bradley B.A., Mooers A.O., Quarmby L.M. (2007). Phylogenetic analysis of the Neks reveals early diversification of ciliary-cell. PLoS ONE.

[9] Pavan I., Peres de Oliveira A., Dias P., Basei F., Issayama L., Ferezin C., Silva F., Rodrigues de Oliveira A., Moura L.A., Martins M. (2021). On Broken Ne(c)ks and Broken DNA: The role of human NEKs in the DNA damage response. Cells..

[10] de Castro Ferezin C., Lim Kam Sian T.C.C., Wu Y., Ma X., Chüeh A.C., Huang C., Schittenhelm R.B., Kobarg J., Daly R.J. (2022). Identification of biological pathways and processes regulated by NEK5 in breast epithelial cells via an integrated proteomic approach. Cell Commun. Signal..

[11] Chen Y., Jiang L., Zhang L., Chi H., Wang Q. (2025). Immune microenvironment and molecular mechanisms in endometrial cancer: Implications for resistance and innovative treatments. Discov. Oncol..

[12] Taylor S.S., Kornev A.P. (2011). Protein kinases: Evolution of dynamic regulatory proteins. Trends Biochem. Sci..

[13] Melo-Hanchuk T.D., Slepicka P.F., Meirelles G.V., Basei F.L., Lovato D.V., Granato D.C., Pauletti B.A., Kobarg J., Palmieri L., Leme A.F.P. (2017). NEK1 kinase domain structure and its dynamic protein interactome after exposure to cisplatin. Sci. Rep..

[14] Bayliss R., Fry A., Haq T., Yeoh S. (2012). On the molecular mechanisms of mitotic kinase activation. Open Biol..

[15] Rellos P., Ivins F.J., Baxter J.E., Pike A., Nott T.J., Parkinson D.M., Das S., Howell S., Fedorov O., Shen Q.Y. (2007). Structure and regulation of the human Nek2 centrosomal kinase. J Biol Chem..

[16] van de Kooij B., Creixell P., van Vlimmeren A., Joughin B.A., Miller C.J., Haider N., Simpson C.D., Linding R., Stambolic V., Turk B.E. (2019). Comprehensive substrate specificity profiling of the human Nek kinome reveals unexpected signaling outputs. eLife.

[17] Richards M.W., O’Regan L., Mas-Droux C., Blot J.M.Y., Cheung J., Hoelder S., Fry A.M., Bayliss R. (2009). An autoinhibitory tyrosine motif in the cell-cycle-regulated Nek7 kinase is released through binding of Nek 9. Mol. Cell.

[18] Haq T., Richards M.W., Burgess S.G., Gallego P., Yeoh S., O’Regan L., Reverter D., Roig J., Fry A.M., Bayliss R. (2015). Mechanistic basis of Nek7 activation through Nek9 binding and induced dimerization. Nat. Commun..

[19] Lupas A.N., Bassler J., Dunin-Horkawicz S. (2017). The structure and topology of α-helical coiled coils. Fibrous Proteins: Structures and Mechanisms.

[20] Croasdale R., Ivins F.J., Muskett F., Daviter T., Scott D.J., Hardy T., Smerdon S.J., Fry A.M., Pfuhl M., Chin J.W. (2011). An undecided coiled coil: The leucine zipper of Nek2 kinase exhibits atypical conformational exchange dynamics. J. Biol. Chem..

[21] Helps N.R., Luo X., Barker H.M., Cohen P.T. (2000). NIMA-related kinase 2 (Nek2), a cell-cycle-regulated protein kinase localized to centrosomes, is complexed to protein phosphatase 1. Biochem. J..

[22] Hames R.S., Wattam S.L., Yamano H., Bacchieri R., Fry A.M. (2001). APC/C-mediated destruction of the centrosomal kinase Nek2A occurs in early mitosis and depends upon a cyclin A-type D-box. EMBO J..

[23] Rechsteiner M., Rogers S.W. (1996). PEST sequences and regulation by proteolysis. Trends Biochem. Sci..

[24] Mendenhall M.D., Hodge A.E. (1998). Regulation of Cdc28 cyclin-dependent protein kinase activity during the cell cycle of the yeast Saccharomyces cerevisiae. Microbiol. Mol. Biol. Rev..

[25] Xu G., Bernaudo S., Fu G., Lee D.Y., Yang B.B., Peng C. (2008). Cyclin G2 is degraded through the ubiquitin-proteasome pathway and mediates the antiproliferative effect of activin receptor-like kinase 7. Mol. Biol. Cell.

[26] Li Y., Zhu J., Zhai F., Kong L., Li H., Jin X. (2024). Advances in the understanding of nuclear pore complexes in human diseases. J. Cancer Res. Clin. Oncol..

[27] Sekhar K.R., Freeman M.L. (1998). PEST sequences in proteins involved in cyclic nucleotide signalling pathways. J. Recept. Signal Transduct. Res..

[28] Burton J.L., Solomon M.J. (2001). D box and KEN box motifs in budding yeast Hsl1p are required for APC-mediated degradation and direct binding to Cdc20p and Cdh1p. Genes Dev..

[29] Bachus S., Graves D., Fulham L., Akkerman N., Stephanson C., Shieh J., Pelka P. (2022). In mitosis you are not: The NIMA family of kinases in *Aspergillus*, yeast, and mammals. Int. J. Mol. Sci..

[30] Chang J., Baloh R.H., Milbrandt J. (2009). The NIMA-family kinase Nek3 regulates microtubule acetylation in neurons. J. Cell Sci..

[31] Krien M.J.E., West R.R., John U.P., Koniaras K., McIntosh J.R., O’Connell M.J. (2002). The fission yeast NIMA kinase Fin1p is required for spindle function and nuclear envelope integrity. EMBO J..

[32] Fuller-Pace F.V. (2013). DEAD box RNA helicase functions in cancer. RNA Biol..

[33] Xing Z., Ma W.K., Tran E.J. (2019). The DDX5/Dbp2 subfamily of DEAD-box RNA helicases. Wiley Interdiscip. Rev. RNA.

[34] Hirth A., Fatti E., Netz E., Acebron S.P., Papageorgiou D., Švorinić A., Cruciat C.-M., Karaulanov E., Gopanenko A., Zhu T. (2024). DEAD box RNA helicases are pervasive protein kinase interactors and activators. Genome Res..

[35] Basei F.L., Silva I.R.E., Firmino Dias P.R., Ferezin C.C., Peres de Oliveira A., Issayama L.K., Moura L.A.R., Riback da Silva F., Kobarg J. (2024). The mitochondrial connection: The Nek kinases’ new functional axis in mitochondrial homeostasis. Cells.

[36] Cargill M., Venkataraman R., Lee S. (2021). DEAD-box RNA helicases and genome stability. Genes.

[37] England J.R., Huang J., Jennings M.J., Makde R.D., Tan S. (2010). RCC1 uses a conformationally diverse loop region to interact with the nucleosome: A model for the RCC1-nucleosome complex. J. Mol. Biol..

[38] Seki T., Hayashi N., Nishimoto T. (1996). RCC1 in the Ran pathway. J. Biochem..

[39] Hadjebi O., Casas-Terradellas E., Garcia-Gonzalo F.R., Rosa J.L. (2008). The RCC1 superfamily: From genes, to function, to disease. Biochim. Biophys. Acta.

[40] Roig J. (2025). NEK8, a NIMA-family protein kinase at the core of the ciliary INV complex. Cell Commun. Signal..

[41] Bertran M.T., Sdelci S., Regué L., Avruch J., Caelles C., Roig J. (2011). Nek9 is a Plk1-activated kinase that controls early centrosome separation through Nek6/7 and Eg 5. EMBO J..

[42] Renault L., Kuhlmann J., Henkel A., Wittinghofer A. (2001). Structural basis for guanine nucleotide exchange on Ran by the regulator of chromosome condensation (RCC1). Cell.

[43] Roig J., Mikhailov A., Belham C., Avruch J. (2002). Nercc1, a mammalian NIMA-family kinase, binds the Ran GTPase and regulates mitotic progression. Genes Dev..

[44] Yang S.W., Gao C., Chen L., Song Y.L., Zhu J.L., Qi S.T., Jiang Z.Z., Li Y.Q., Han C.S., Sun Q.Y. (2012). Nek9 regulates spindle organization and cell cycle progression during mouse oocyte meiosis and its location in early embryo mitosis. Cell Cycle.

[45] Li H., Li J., Zhang Y., Cao R., Guo C., Jiao M. (2025). The NIMA-related kinase family and cancer. Front. Oncol..

[46] Huber A.H., Nelson W.J., Weis W.I. (1997). Three-dimensional structure of the armadillo repeat region of β-catenin. Cell..

[47] Arrías P.N., Osmanli Z., Paralta E., Chinestrad P.M., Monzon A.M., Tosatto S.C.E. (2024). Diversity and structural-functional insights of alpha-solenoid proteins. Prot. Sci..

[48] Power K.M., Akella J.S., Gu A., Walsh J.D., Bellotti S., Morash M., Zhang W., Ramadan Y.H., Ross N., Golden A. (2020). Mutation of NEKL-4/NEK10 and TTLL genes suppress neuronal ciliary degeneration caused by loss of CCPP-1 deglutamylase function. PLoS Genet..

[49] Haider N., Dutt P., van de Kooij B., Ho J., Palomero L., Pujana M.A., Yaffe M., Stambolic V. (2020). NEK10 tyrosine phosphorylates p53 and controls its transcriptional activity. Oncogene.

[50] Meirelles G.V., Perez A.M., de Souza E.E., Basei F.L., Papa P.F., Melo Hanchuk T.D., Cardoso V.B., Kobarg J. (2014). “Stop Ne(c)king around”: How interactomics contributes to functionally characterize Nek family kinases. World J. Biol. Chem..

[51] Maier B.D., Petursson B., Lussana A., Petsalaki E. (2025). Data-driven extraction of human kinase-substrate relationships from Omics datasets. Mol. Cell Proteomics..

[52] Chivukula R.R., Montoro D.T., Leung H.M., Yang J., Shamseldin H.E., Taylor M.S., Dougherty G.W., Zariwala M.A., Carson J., Daniels M.L.A. (2020). A human ciliopathy reveals essential functions for NEK10 in airway mucociliary clearance. Nat. Med..

[53] Fry A.M., Arnaud L., Nigg E.A. (1999). Activity of the human centrosomal kinase, Nek2, depends on an unusual leucine zipper dimerization motif. ” J. Biol. Chem..

[54] Dutt P., Haider N., Mouaaz S., Stambolic V. (2024). β-Catenin turnover is regulated by Nek10-mediated tyrosine phosphorylation in A549 lung adenocarcinoma cells. Proc. Natl. Acad. Sci. USA.

[55] Fry A.M., Bayliss R., Roig J. (2017). Mitotic regulation by NEK kinase networks. Front. Cell Dev. Biol..

[56] Freund I., Hehlgans S., Martin D., Ensminger M., Fokas E., Rödel C., Löbrich M., Rödel F. (2020). Fractionation-dependent radiosensitization by molecular targeting of Nek1. Cells.

[57] Singh V., Connelly Z.M., Shen X., De Benedetti A. (2017). Identification of the proteome complement of human TLK1 reveals it binds and phosphorylates NEK1 regulating its activity. Cell Cycle.

[58] Maréchal A., Zou L. (2013). DNA damage sensing by the ATM and ATR kinases. Cold Spring Harb Perspect Biol..

[59] Liu S., Ho C.K., Ouyang J., Zou L. (2013). Nek1 kinase associates with ATR-ATRIP and primes ATR for efficient DNA damage signaling. Proc. Natl. Acad. Sci. USA.

[60] Cannavo E., Giordano Reginato M., Cejka P. (2019). Stepwise 5′ DNA end-specific resection of DNA breaks by the Mre11-Rad50-Xrs2 and Sae2 nuclease ensemble. Proc. Natl. Acad. Sci. USA.

[61] Spies J., Waizenegger A., Barton O., Sürder M., Wright W.D., Heyer W.D., Löbrich M. (2016). Nek1 regulates Rad54 to orchestrate homologous recombination and replication fork stability. Mol. Cell.

[62] Gregorczyk M., Pastore G., Muñoz I., Carroll T., Streubel J., Munro M., Lis P., Lange S., Lamoliatte F., Macartney T. (2023). *;* et al. Functional characterization of C21ORF2 association with the NEK1 kinase mutated in human diseases. Life Sci. Alliance.

[63] Watanabe Y., Nakagawa T., Akiyama T., Nakagawa M., Suzuki N., Warita H., Aoki M., Nakayama K. (2020). An amyotrophic lateral sclerosis-associated mutant of C21ORF2 is stabilized by NEK1-mediated hyperphosphorylation and the inability to bind FBXO3. iScience.

[64] Hardy T., Lee M., Hames R.S., Prosser S.L., Cheary D.M., Samant M.D., Schultz F., Baxter J.E., Rhee K., Fry A.M. (2014). Multisite phosphorylation of C-Nap1 releases it from Cep135 to trigger centrosome disjunction. J. Cell Sci..

[65] Bahe S., Stierhof Y.D., Wilkinson C.J., Leiss F., Nigg E.A. (2005). Rootletin forms centriole-associated filaments and functions in centrosome cohesion. J. Cell Biol..

[66] Wei R., Ngo B., Wu G., Lee W.H. (2011). Phosphorylation of the Ndc80 complex protein, HEC1, by Nek2 kinase modulates chromosome alignment and signaling of the spindle assembly checkpoint. Mol. Biol. Cell..

[67] Fang Y., Zhang X. (2016). Targeting NEK2 as a promising therapeutic approach for cancer treatment. Cell Cycle.

[68] Park J., Rhee K. (2013). NEK2 phosphorylation antagonizes the microtubule stabilizing activity of centrobin. Biochem. Biophys. Res. Commun..

[69] Mbom B.C., Siemers K.A., Ostrowski M.A., Nelson W.J., Barth A.I. (2014). Nek2 phosphorylates and stabilizes β-catenin at mitotic centrosomes downstream of Plk1. Mol Biol Cell..

[70] Miller S.L., Antico G., Raghunath P.N., Tomaszewski J.E., Clevenger C.V. (2007). Nek3 kinase regulates prolactin-mediated cytoskeletal reorganization and motility of breast cancer cells. Oncogene.

[71] Nguyen C.L., Possemato R., Bauerlein E.L., Xie A., Scully R., Hahn W.C. (2012). Nek4 regulates entry into replicative senescence and the response to DNA damage in human fibroblasts. Mol. Cell. Biol..

[72] Ferezin C.D.C., Basei F.L., Melo-Hanchuk T.D., de Oliveira A.L., Peres de Oliveira A., Mori M.P., de Souza-Pinto N.C., Kobarg J. (2021). NEK5 interacts with LonP1 and its kinase activity is essential for the regulation. FEBS Open Bio..

[73] Adib R., Montgomery J.M., Atherton J., O’Regan L., Richards M.W., Straatman K.R., Roth D., Straube A., Bayliss R., Moores C.A. (2019). Mitotic phosphorylation by NEK6 and NEK7 reduces the microtubule affinity of EML4 to promote chromosome congression. Sci. Signal..

[74] Rapley J., Nicolàs M., Groen A., Regué L., Bertran M.T., Caelles C., Avruch J., Roig J. (2008). The NIMA-family kinase Nek6 phosphorylates the kinesin Eg5 at a novel site necessary for mitotic spindle formation. J. Cell Sci..

[75] Hashimoto Y., Akita H., Hibino M., Kohri K., Nakanishi M. (2002). Identification and characterization of Nek6 protein kinase, a potential human homolog of NIMA histone H3 kinase. Biochem. Biophys. Res. Commun..

[76] O’Regan L., Sampson J., Richards M.W., Knebel A., Roth D., Hood F.E., Straube A., Royle S.J., Bayliss R., Fry A.M. (2015). Hsp72 is targeted to the mitotic spindle by Nek6 to promote K-fiber assembly and mitotic progression. J. Cell Biol..

[77] Takatani S., Ozawa S., Yagi N., Hotta T., Hashimoto T., Takahashi Y., Takahashi T., Motose H. (2017). Directional cell expansion requires NIMA-related kinase 6 (NEK6)-mediated cortical microtubule destabilization. Sci. Rep..

[78] Belham C., Comb M.J., Avruch J. (2001). Identification of the NIMA family kinases NEK6/7 as regulators of the p70 ribosomal S6 kinase. Curr. Biol..

[79] Jeon Y.J., Lee K.Y., Cho Y.Y., Pugliese A., Kim H.G., Jeong C.H., Bode A.M., Dong Z. (2010). Role of NEK6 in tumor promoter-induced transformation in JB6 C141 mouse skin epidermal cells. J. Biol. Chem..

[80] Tan R., Nakajima S., Wang Q., Sun H., Xue J., Wu J., Hellwig S., Zeng X., Yates N.A., Smithgall T.E. (2017). Nek7 Protects Telomeres from Oxidative DNA Damage by Phosphorylation and Stabilization of TRF1. Mol. Cell.

[81] Xu J., Zhang L., Duan Y., Sun F., Odeh N., He Y., Núñez G. (2025). NEK7 phosphorylation amplifies NLRP3 inflammasome activation downstream of potassium efflux and gasdermin D. Sci. Immunol..

[82] Freixo F., Martinez Delgado P., Manso Y., Sánchez-Huertas C., Lacasa C., Soriano E., Roig J., Lüders J. (2018). NEK7 regulates dendrite morphogenesis in neurons via Eg5-dependent microtubule stabilization. Nat. Commun..

[83] Czarnecki P.G., Gabriel G.C., Manning D.K., Sergeev M., Lemke K., Klena N.T., Liu X., Chen Y., Li Y., San Agustin J.T. (2015). ANKS6 is the critical activator of NEK8 kinase in embryonic situs determination and organ patterning. Nat. Commun..

[84] Cao B., Zhang K., Pan C., Dong Y., Lu F. (2023). NEK8 regulates colorectal cancer progression via phosphorylating MYC. Cell Commun. Signal..

[85] Holland P.M., Milne A., Garka K., Johnson R.S., Willis C., Sims J.E., Rauch C.T., Bird T.A., Virca G.D. (2002). Purification, cloning, and characterization of Nek8, a novel NIMA-related kinase, and its candidate substrate Bicd2. J. Biol. Chem..

[86] Sun Z., Wang Q., Sun L., Wu M., Li S., Hua H., Sun Y., Ni T., Zhou C., Huang S. (2022). Acetaminophen-induced reduction of NIMA-related kinase 7 expression exacerbates acute liver injury. JHEP Rep..

[87] Sdelci S., Schütz M., Pinyol R., Bertran M.T., Regué L., Caelles C., Vernos I., Roig J. (2012). Nek9 phosphorylation of NEDD1/GCP-WD contributes to Plk1 control of γ-tubulin recruitment to the mitotic centrosome. Curr. Biol..

[88] Lu G., Du R., Dong J., Sun Y., Zhou F., Feng F., Feng B., Han Y., Shang Y. (2023). Cancer-associated fibroblast derived SLIT2 drives gastric cancer cell metastasis by activating NEK9. Cell Death Dis..

[89] Lu G., Tian S., Sun Y., Dong J., Wang N., Zeng J., Nie Y., Wu K., Han Y., Feng B. (2021). NEK9, a novel effector of IL-6/STAT3, regulates metastasis of gastric cancer by targeting ARHGEF2 phosphorylation. Theranostics.

[90] Gallisà-Suñé N., Sànchez-Fernàndez-de-Landa P., Zimmermann F., Serna M., Regué L., Paz J., Llorca O., Lüders J., Roig J. (2023). BICD2 phosphorylation regulates dynein function and centrosome separation in G2 and M. Nat. Commun..

[91] Melixetian M., Klein D.K., Sørensen C.S., Helin K. (2009). NEK11 regulates CDC25A degradation and the IR-induced G_2_/M checkpoint. Nat. Cell Biol..

[92] Sørensen C.S., Melixetian M., Klein D.K., Helin K. (2010). NEK11: Linking CHK1 and CDC25A in DNA damage checkpoint signaling. Cell Cycle.

[93] Fang X., Lin H., Wang X., Zuo Q., Qin J., Zhang P. (2015). The NEK1 interactor, C21ORF2, is required for efficient DNA damage repair. Acta Biochim. Biophys. Sin..

[94] Chen Y., Chen C.-F., Chiang H.-C., Pena M., Polci R., Wei R.L., Edwards R.A., Hansel D.E., Chen P.-L., Riley D.J. (2011). Mutation of NIMA-related kinase 1 (NEK1) leads to chromosome instability. Mol. Cancer.

[95] Mann J.R., McKenna E.D., Mawrie D., Papakis V., Alessandrini F., Anderson E.N., Mayers R., Ball H.E., Kaspi E., Lubinski K. (2023). Loss of function of the ALS-associated NEK1 kinase disrupts microtubule homeostasis and nuclear import. Sci. Adv..

[96] Noh M.-Y., Oh S.-i., Kim Y.-E., Cha S.J., Sung W., Oh K.-W., Park Y., Mun J.Y., Ki C.-S., Nahm M. (2025). Mutations in NEK1 cause ciliary dysfunction as a novel pathogenic mechanism in amyotrophic lateral sclerosis. Mol. Neurodegener..

[97] Wang H., Qi W., Zou C., Xie Z., Zhang M., Naito M.G., Mifflin L., Liu Z., Najafov A., Pan H. (2021). NEK1-mediated retromer trafficking promotes blood–brain barrier integrity by regulating glucose metabolism and RIPK1 activation. Nat. Commun..

[98] Awuah W.A., Tan J.K., Shkodina A.D., Ferreira T., Adebusoye F.T., Mazzoleni A., Wellington J., David L., Chilcott E., Huang H. (2023). Hereditary spastic paraplegia: Novel insights into the pathogenesis and management. SAGE Open Med..

[99] Martins M.B., Perez A.M., Bohr V.A., Wilson D.M., Kobarg J. (2021). NEK1 deficiency affects mitochondrial functions and the transcriptome of key DNA repair pathways. Mutagenesis..

[100] Thiel C., Kessler K., Giessl A., Dimmler A., Shalev S.A., von der Haar S., Zenker M., Zahnleiter D., Stöss H., Beinder E. (2011). NEK1 mutations cause short-rib polydactyly syndrome type Majewski. Am. J. Hum. Genet..

[101] He Q., Zhou Y., Jin J., Tian Q., Li H., Hou B., Xie A. (2024). Association between *NEK1* gene polymorphisms and the potential risk of sporadic Parkinson’s disease in the Chinese northern Han population: A case-control study. Neurosci. Lett..

[102] Shalom O., Shalva N., Altschuler Y., Motro B. (2008). The mammalian Nek1 kinase is involved in primary cilium formation. FEBS Lett..

[103] White M.C., Quarmby L.M. (2008). The NIMA-family kinase, Nek1 affects the stability of centrosomes and ciliogenesis. BMC Cell Biol..

[104] Chen Y., Chiang H.C., Litchfield P., Pena M., Juang C., Riley D.J. (2014). Expression of Nek1 during kidney development and cyst formation in multiple nephron segments in the Nek1-deficient kat2J mouse model of polycystic kidney disease. J Biomed Sci..

[105] Wang W., Wu T., Kirschner M.W. (2014). The master cell cycle regulator APC-Cdc20 regulates ciliary length and disassembly of the primary cilium. eLife.

[106] Zhu J., Cai Y., Liu P., Zhao W. (2016). Frequent Nek1 overexpression in human gliomas. Biochem. Biophys. Res. Commun..

[107] Khalil M.I., De Benedetti A. (2022). Tousled-like kinase 1: A novel factor with multifaceted role in mCRPC progression and development of therapy resistance. Cancer Drug Resist..

[108] Polci R., Peng A., Chen P.L., Riley D.J., Chen Y. (2004). NIMA-related protein kinase 1 is involved early in the ionizing radiation-induced DNA damage response. Cancer Res..

[109] Dyrskjøt L., Reinert T., Algaba F., Christensen E., Nieboer D., Hermann G.G., Mogensen K., Beukers W., Marquez M., Segersten U. (2017). Prognostic impact of a 12-gene progression score in non-muscle-invasive bladder cancer: A prospective multicentre validation study. Eur. Urol..

[110] Lindskrog S.V., Prip F., Lamy P., Taber A., Groeneveld C.S., Birkenkamp-Demtröder K., Jensen J.B., Strandgaard T., Nordentoft I., Christensen E. (2021). An integrated multi-Omics analysis identifies prognostic molecular subtypes of non-muscle-invasive bladder cancer. Nat. Commun..

[111] Rapley J., Baxter J.E., Blot J., Wattam S.L., Casenghi M., Meraldi P., Nigg E.A., Fry A.M. (2005). Co-ordinate regulation of the mother centriole component Nlp by Nek2 and Plk1 protein kinases. Mol. Cell. Biol..

[112] Mardin B.R., Agircan F.G., Lange C., Schiebel E. (2011). Plk1 controls the Nek2A-PP1γ antagonism in centrosome disjunction. Curr. Biol..

[113] Mayor T., Hacker U., Stierhof Y.D., Nigg E.A. (2002). The mechanism regulating the dissociation of the centrosomal protein C-Nap1 from mitotic spindle poles. J. Cell Sci..

[114] Chen Y., Riley D.J., Zheng L., Chen P.L., Lee W.H. (2002). Phosphorylation of the mitotic regulator protein Hec1 by Nek2 kinase is essential for faithful chromosome segregation. J. Biol. Chem..

[115] Liu S.T., Zhang H. (2016). The Mitotic Checkpoint Complex (MCC): Looking Back and Forth after 15 Years. AIMS Mol. Sci..

[116] DeLuca J.G., Gall W.E., Ciferri C., Cimini D., Musacchio A., Salmon E.D. (2006). Kinetochore microtubule dynamics and attachment stability are regulated by Hec1. Cell..

[117] Sundin L.J., Guimaraes G.J., DeLuca J.G. (2011). The NDC80 complex proteins Nuf2 and Hec1 make distinct contributions to kinetochore-microtubule attachment in mitosis. Mol. Biol. Cell.

[118] Nguyen K., Boehling J., Tran M.N., Cheng T., Rivera A., Collins-Burow B.M., Lee S.B., Drewry D.H., Burow M.E. (2023). NEK family review and correlations with patient survival outcomes in various cancer types. Cancers.

[119] Faragher A.J., Fry A.M. (2003). Nek2A kinase stimulates centrosome disjunction and is required for formation of bipolar mitotic spindles. Mol. Biol. Cell.

[120] Wu J., Luo D., Tou L., Xu H., Jiang C., Wu D., Que H., Zheng J. (2025). NEK2 affects the ferroptosis sensitivity of gastric cancer cells by regulating the expression of HMOX1 through Keap1/Nrf 2. Mol. Cell. Biochem..

[121] Franqui-Machin R., Hao M., Bai H., Gu Z., Zhan X., Habelhah H., Jethava Y., Qiu L., Frech I., Tricot G. (2018). Destabilizing NEK2 overcomes resistance to proteasome inhibition in multiple myeloma. J. Clin. Investig..

[122] Howes S.C., Alushin G.M., Shida T., Nachury M.V., Nogales E. (2014). Effects of tubulin acetylation and tubulin acetyltransferase binding on microtubule structure. Mol. Biol. Cell..

[123] Zhang Y., Chen W., Zeng W., Lu Z., Zhou X. (2020). Biallelic loss of function NEK3 mutations deacetylate α-tubulin and downregulate NUP205 that predispose individuals to cilia-related abnormal cardiac left-right patterning. Cell Death Dis..

[124] Soppina V., Herbstman J.F., Skiniotis G., Verhey K.J. (2012). Luminal localization of α-tubulin K40 acetylation by cryo-EM analysis of fab-labeled microtubules. PLoS ONE.

[125] Chen Y., Zhou G., Yu M. (2025). Conformational dynamics of the nuclear pore complex central channel. Biochem. Soc. Trans..

[126] Basei F.L., de Castro Ferezin C., Rodrigues de Oliveira A.L., Muñoz J.P., Zorzano A., Kobarg J. (2022). Nek4 regulates mitochondrial respiration and morphology. FEBS J..

[127] Ding N.H., Zhang L., Xiao Z., Rong Z.X., Li Z., He J., Chen L., Ou D.M., Liao W.H., Sun L.Q. (2018). NEK4 kinase regulates EMT to promote lung cancer metastasis. J. Cell. Mol. Med..

[128] Thiery J.P., Acloque H., Huang R.Y.J., Nieto M.A. (2009). Epithelial-mesenchymal transitions in development and disease. Cell..

[129] Park S.J., Jo D.S., Jo S.-Y., Shin D.W., Shim S., Jo Y.K., Shin J.H., Ha Y.J., Jeong S.-Y., Hwang J.J. (2016). Inhibition of never in mitosis A (NIMA)-related kinase-4 reduces survivin expression and sensitizes cancer cells to TRAIL-induced cell death. Oncotarget..

[130] Chandra D., Liu J.W., Tang D.G. (2002). Early mitochondrial activation and cytochrome c up-regulation during apoptosis. J. Biol. Chem..

[131] Wang C., Youle R.J. (2009). The role of mitochondria in apoptosis. Annu. Rev. Genet..

[132] Jiang X., Wang X. (2004). Cytochrome C-mediated apoptosis. Annu Rev Biochem..

[133] Ambrosini G., Adida C., Altieri D.C. (1998). IAP-family protein survivin inhibits caspase activity and apoptosis induced by Fas (CD95), Bax, caspases, and anticancer drugs. Cancer Res..

[134] Doles J., Hemann M.T. (2010). Nek4 status differentially alters sensitivity to distinct microtubule poisons. Cancer Res..

[135] Abouzeid H.A., Kassem L., Liu X., Abuelhana A. (2025). Paclitaxel resistance in breast cancer: Current challenges and recent advanced therapeutic strategies. Cancer Treat. Res. Commun..

[136] Xiao H., Verdier-Pinard P., Fernandez-Fuentes N., Burd B., Angeletti R., Fiser A., Horwitz S.B., Orr G.A. (2006). Insights into the mechanism of microtubule stabilization by Taxol. Proc. Natl. Acad. Sci. USA.

[137] Prosser S.L., Sahota N.K., Pelletier L., Morrison C.G., Fry A.M. (2015). Nek5 promotes centrosome integrity in interphase and loss of centrosome cohesion in mitosis. J. Cell Biol..

[138] Sigal Y.M., Zhou R., Zhuang X. (2018). Visualizing and discovering cellular structures with super-resolution microscopy. Science..

[139] Matossian M.D., Wells C.I., Zuercher W.J., Collins-Burow B.M., Drewry D.H., Burow M.E. (2021). NEK5 activity regulates the mesenchymal and migratory phenotype in breast cancer cells. Breast Cancer Res. Treatment..

[140] Zhou X., Nie H., Wang C., Yu X., Yang X., He X., Ou C. (2024). Prognostic value and therapeutic potential of NEK family in stomach adenocarcinoma. J. Cancer.

[141] Nusinow D.P., Szpyt J., Ghandi M., Rose C.M., McDonald E.R., Kalocsay M., Jané-Valbuena J., Gelfand E., Schweppe D.K., Jedrychowski M. (2020). Quantitative proteomics of the cancer cell line encyclopedia. Cell..

[142] Li T., Fan J., Wang B., Traugh N., Chen Q., Liu J.S., Li B., Liu X.S. (2017). TIMER: A web server for comprehensive analysis of tumor-infiltrating immune cells. Cancer Res..

[143] Yu Y., Shen T., Zhong X., Wang L.-L., Tai W., Zou Y., Qin J., Zhang Z., Zhang C.-L. (2021). NEK6 is an injury-responsive kinase cooperating with STAT3 in regulation of reactive astrogliosis. Glia.

[144] Hu X., li J., Fu M., Wang W. (2021). The JAK/STAT signaling pathway: From bench to clinic. Sig Transduct Target Ther..

[145] Peron M., Dinarello A., Meneghetti G., Martorano L., Betto R.M., Facchinello N., Tesoriere A., Tiso N., Martello G., Argenton F. (2021). Y705 and S727 are required for the mitochondrial import and transcriptional activities of STAT3, and for regulation of stem cell proliferation. Development.

[146] Zhu M., Sun Y., Xue H., Wu G., Wang Z., Shi J., Ma J., Gu B., Yan X. (2023). NEK6 promotes the progression of osteosarcoma through activating STAT3 signaling pathway by down-regulation of miR-26a-5p. Int. J. Gen. Med..

[147] Pavan I.C.B., Basei F.L., Brandemarte M., Rosa e Silva I., Issayama L.K., Mancini M.C.S., Goís M.M., da Silva L.G.S., Bezerra R.M.N., Simabuco F.M. (2023). NEK6 Regulates Redox Balance and DNA Damage Response in DU-145 Prostate Cancer Cells. Cells..

[148] De Donato M., Fanelli M., Mariani M., Raspaglio G., Pandya D., He S., Fiedler P., Petrillo M., Scambia G., Ferlini C. (2015). Abstract 4327: Nek6 and Hif-1α cooperate with the cytoskeletal gateway of drug resistance to drive outcome in serous ovarian cancer. Cancer Res..

[149] Carmeliet P., Dor Y., Herbert J.-M., Fukumura D., Brusselmans K., Dewerchin M., Neeman M., Bono F., Abramovitch R., Maxwell P. (1998). Role of HIF-1α in hypoxia-mediated apoptosis, cell proliferation and tumour angiogenesis. Nature..

[150] He Y., Zeng M.Y., Yang D., Motro B., Nunez G. (2016). NEK7 is an essential mediator of NLRP3 activation downstream of potassium efflux. Nature.

[151] He H., Jiang H., Chen Y., Ye J., Wang A., Wang C., Liu Q., Liang G., Deng X., Jiang W. (2018). Oridonin is a covalent NLRP3 inhibitor with strong anti-inflammasome activity. Nat. Commun..

[152] Kelley N., Jeltema D., Duan Y., He Y. (2019). The NLRP3 inflammasome: An overview of mechanisms of activation and regulation. Int. J. Mol. Sci..

[153] Yan Z., Da Q., Li Z., Lin Q., Yi J., Su Y., Yu G., Ren Q., Liu X., Lin Z. (2022). Inhibition of NEK7 suppressed hepatocellular carcinoma progression by mediating cancer cell pyroptosis. Front. Oncol..

[154] Li G., Dong Y., Liu D., Zou Z., Hao G., Gao X., Pan P., Liang G. (2020). NEK7 coordinates rapid neuroinflammation after subarachnoid hemorrhage in mice. Front. Neurol..

[155] Sharif H., Wang L., Wang W.L., Magupalli V.G., Andreeva L., Qiao Q., Hauenstein A.V., Wu Z., Núñez G., Mao Y. (2019). Structural mechanism for NEK7-licensed activation of NLRP3 inflammasome. Nature..

[156] Kim S.K., Choe J.Y., Park K.Y. (2019). Anti-inflammatory effect of artemisinin on uric acid-induced NLRP3 inflammasome activation through blocking interaction between NLRP3 and NEK7. Biochem. Biophys. Res. Commun..

[157] Chen Y., Meng J., Bi F., Li H., Chang C., Ji C., Liu W. (2019). NEK7 regulates NLRP3 inflammasome activation and neuroinflammation post-traumatic brain injury. Front. Mol. Neurosci..

[158] Li D., Wang L., Ou J., Wang C., Zhou J., Lu L., Wu Y., Gao J. (2021). Reactive oxygen species induced by uric acid promote NRK-52E cell apoptosis through the NEK7-NLRP3 signaling pathway. Mol. Med. Rep..

[159] Seok J.K., Kang H.C., Cho Y.Y., Lee H.S., Lee J.Y. (2021). Therapeutic regulation of the NLRP3 inflammasome in chronic inflammatory diseases. Arch. Pharm. Res..

[160] Chen Y., Ye X., Escames G., Lei W., Zhang X., Li M., Jing T., Yao Y., Qiu Z., Wang Z. (2023). The NLRP3 inflammasome: Contributions to inflammation-related diseases. Cell Mol. Biol. Lett..

[161] Yao J., Sterling K., Wang Z., Zhang Y., Song W. (2024). The role of inflammasomes in human diseases and their potential as therapeutic targets. Signal Transduct. Target Ther..

[162] Lui K., Huang Y. (2009). RanGTPase: A key regulator of nucleocytoplasmic trafficking. Mol Cell Pharmacol..

[163] Kalab P., Heald R. (2008). The RanGTP gradient—a GPS for the mitotic spindle. J Cell Sci..

[164] Zhang Q., Han X., Chen J., Xie X., Xu J., Zhao Y., Shen J., Hu L., Xu P., Song H. (2018). Yes-associated protein (YAP) and transcriptional coactivator with PDZ-binding motif (TAZ) mediate cell density-dependent proinflammatory responses. J. Biol. Chem..

[165] Choi H.J.C., Lin J.-R., Vannier J.-B., Slaats G.G., Kile A.C., Paulsen R.D., Manning D.K., Beier D.R., Giles R.H., Boulton S.J. (2013). NEK8 links the ATR-regulated replication stress response and S phase CDK activity to renal ciliopathies. Mol. Cell..

[166] Otto E.A., Trapp M.L., Schultheiss U.T., Helou J., Quarmby L.M., Hildebrandt F. (2008). NEK8 mutations affect ciliary and centrosomal localization and may cause nephronophthisis. J. Am. Soc. Nephrol..

[167] Shiba D., Manning D.K., Koga H., Beier D.R., Yokoyama T. (2010). Inv acts as a molecular anchor for Nphp3 and Nek8 in the proximal segment of primary cilia. Cytoskeleton.

[168] Grampa V., Delous M., Zaidan M., Odye G., Thomas S., Elkhartoufi N., Filhol E., Niel O., Silbermann F., Lebreton C. (2016). Novel NEK8 mutations cause severe syndromic renal cystic dysplasia through YAP dysregulation. PLoS Genet..

[169] Abeyta A., Castella M., Jacquemont C., Taniguchi T. (2017). NEK8 regulates DNA damage-induced RAD51 foci formation and replication fork protection. Cell Cycle..

[170] Kang E., Kim H.K., Lee H.B., Han W. (2023). Never in mitosis gene A-related kinase-8 promotes proliferation, migration, invasion, and stemness of breast cancer cells via β-catenin signalling activation. Sci. Rep..

[171] Bowers A.J., Boylan J.F. (2004). Nek8, a NIMA family kinase member, is overexpressed in primary human breast tumors. Gene.

[172] Yamamoto Y., Chino H., Tsukamoto S., Ode K.L., Ueda H.R., Mizushima N. (2021). NEK9 regulates primary cilia formation by acting as a selective autophagy adaptor for MYH9/Myosin IIA. Nat. Commun..

[173] Smith S.C., Petrova A.V., Madden M.Z., Wang H., Pan Y., Warren M.D., Hardy C.W., Liang D., Liu E.A., Robinson M.H. (2014). A gemcitabine sensitivity screen identifies a role for NEK9 in the replication stress response. Nucleic Acids Res..

[174] Tan B.C., Lee S.C. (2004). Nek9, a novel FACT-associated protein, modulates interphase progression. J Biol Chem..

[175] Roig J., Groen A., Caldwell J., Avruch J. (2005). Active Nercc1 protein kinase concentrates at centrosomes early in mitosis and is necessary for proper spindle assembly. Mol. Biol. Cell.

[176] Kaneta Y., Ullrich A. (2013). NEK9 depletion induces catastrophic mitosis by impairment of mitotic checkpoint control and spindle dynamics. Biochem. Biophys. Res. Commun..

[177] O’Regan L., Barone G., Adib R., Woo C.G., Jeong H.J., Richardson E.L., Richards M.W., Muller P.A.J., Collis S.J., Fennell D.A. (2020). EML4-ALK V3 oncogenic fusion proteins promote microtubule stabilization and accelerated migration through NEK9 and NEK7. J. Cell Sci..

[178] de Oliveira A.P., Basei F.L., Slepicka P.F., de Castro Ferezin C., Melo-Hanchuk T.D., de Souza E.E., Lima T.I., dos Santos V.T., Mendes D., Silveira L.R. (2020). NEK10 interactome and depletion reveal new roles in mitochondria. Proteome Sci..

[179] de Oliveira A.P., Navarro C.D.C., Dias P.R.F., Arguello T., Walker B.R., Bacman S.R., Sousa L.M., Castilho R.F., Consonni S.R., Moraes C.T. (2024). NEK10 kinase ablation affects mitochondrial morphology, function and protein phosphorylation status. Proteome Sci..

[180] Guo L., Wang Z.B., Wang H.H., Zhang T., Qi S.T., Ouyang Y.C., Sun Q.Y. (2016). Nek11 regulates asymmetric cell division during mouse oocyte meiotic maturation. Biochem. Biophys. Res. Commun..

[181] Minoguchi S., Minoguchi M., Yoshimura A. (2003). Differential control of the NIMA-related kinases, Nek6 and Nek7, by serum stimulation. Biochem. Biophys. Res. Commun..

[182] Panchal N.K., Mohanty S., Prince S.E. (2023). NIMA-related kinase-6 (NEK6) as an executable target in cancer. Clin. Transl. Oncol..

[183] Branon T.C., Bosch J.A., Sanchez A.D., Udeshi N.D., Svinkina T., Carr S.A., Feldman J.L., Perrimon N., Ting A.Y. (2018). Efficient proximity labeling in living cells and organisms with TurboID. Nat Biotechnol..

[184] May D.G., Scott K.L., Campos A.R., Roux K.J. (2020). Comparative application of BioID and TurboID for protein-proximity biotinylation. Cells..

[185] Newman R.H., Zhang J., Zhu H. (2014). Toward a systems level view of dynamic phosphorylation networks. Front. Genet..

[186] Zhu H., Cox E., Qian J. (2012). Functional protein microarray as molecular decathlete: A versatile player in clinical proteomics. Proteom. Clin. Appl..

[187] Newman R.H., Hu J., Rho H.S., Xie Z., Woodard C., Neiswinger J., Cooper C., Shirley M., Clark H.M., Hu S. (2013). Construction of human activity-based phosphorylation networks. Mol. Syst. Biol..

[188] Jonić S. (2016). Cryo-electron Microscopy Analysis of Structurally Heterogeneous Macromolecular Complexes. Comput. Struct. Biotechnol. J..

[189] Abramson J., Adler J., Dunger J., Evans R., Green T., Pritzel A., Ronneberger O., Willmore L., Ballard A.J., Bambrick J. (2024). Accurate structure prediction of biomolecular interactions with AlphaFold 3. Nature..

[190] Hu C.-M., Zhu J., Guo X.E., Chen W., Qiu X.L., Ngo B., Chien R., Wang Y.V., Tsai C.Y., Wu G. (2015). Novel small molecules disrupting Hec1/Nek2 interaction ablate tumor progression by triggering Nek2 degradation through a death-trap mechanism. Oncogene..

[191] Greenwald E.C., Mehta S., Zhang J. (2018). Genetically Encoded Fluorescent Biosensors Illuminate the Spatiotemporal Regulation of Signaling Networks. Chem. Rev..

[192] Newman R.H., Fosbrink M., Zhang J. (2011). Fluorescent biosensors for tracking signaling dynamics in living cells. Chem. Rev..

[193] Newman R.H., Zhang J. (2017). Integrated strategies to gain a systems-level view of dynamic signaling networks. Methods Enzymol..

[194] Nijenhuis W., van Grinsven M.M.P., Kapitein L.C. (2020). An optimized toolbox for the optogenetic control of intracellular transport. J. Cell Biol..

[195] Natsume T., Kanemaki M.T. (2017). Conditional degrons for controlling protein expression at the protein level. Annu. Rev. Genet..

[196] Han X., Sun Y. (2023). PROTACs: A novel strategy for cancer drug discovery and development. MedComm.

